# Remodeling of the Mouse Liver and Skeletal Muscle Metabolome in Response to Continuous Acute Exercise and Disruption of AMPK-Glycogen Interactions

**DOI:** 10.3390/metabo16030205

**Published:** 2026-03-20

**Authors:** Mehdi R. Belhaj, David I. Broadhurst, Thomas Dignan, Jamie Whitfield, Lisa Murray-Segal, Naomi X. Y. Ling, Jonathan S. Oakhill, Bruce E. Kemp, John A. Hawley, Stacey N. Reinke, Nolan J. Hoffman

**Affiliations:** 1 Centre for Human Metabolism and Performance, Mary MacKillop Institute for Health Research, Australian Catholic University, Melbourne, VIC 3002, Australia; mehdi.belhaj@acu.edu.au (M.R.B.); bkemp@svi.edu.au (B.E.K.); john.hawley@acu.edu.au (J.A.H.); 2 School of Science, Edith Cowan University, Joondalup, WA 6027, Australia; d.broadhurst@ecu.edu.au (D.I.B.); t.dignan@ecu.edu.au (T.D.); 3 Metabolic Signalling Laboratory, St. Vincent’s Institute of Medical Research, Fitzroy, VIC 3065, Australia; lmsegal@svi.edu.au (L.M.-S.); nling@svi.edu.au (N.X.Y.L.); joakhill@svi.edu.au (J.S.O.); 4 Department of Medicine, University of Melbourne, Parkville, VIC 3052, Australia; 5 Protein Chemistry and Metabolism, St. Vincent’s Institute of Medical Research, Fitzroy, VIC 3065, Australia; 6 Department of Sport and Exercise Sciences, Institute of Sport, Manchester Metropolitan University, Manchester M1 7EL, UK

**Keywords:** exercise metabolism, untargeted metabolomics, LC-MS, transgenic mouse, AMP-activated protein kinase, mitochondrial respiration

## Abstract

**Background/Objectives**: Acute exercise remodels many interconnected biochemical pathways in metabolically active tissues. This remodeling involves the activation of the energy-sensing AMP-activated protein kinase (AMPK) to maintain cellular energy homeostasis. Critical energy reserves of glycogen, primarily stored in liver and skeletal muscle and known to interact with AMPK, are utilized to help meet increased energy demands with exercise. However, the breadth of metabolic pathways regulated by acute exercise and AMPK’s interactive roles with glycogen remain incompletely understood. This study therefore aimed to map mouse liver and skeletal muscle metabolite responses to continuous acute exercise and disruption of AMPK-glycogen interactions. **Methods**: Liquid chromatography–mass spectrometry-based untargeted metabolomics was used to measure the relative abundance of liver and gastrocnemius muscle metabolites at rest and following an acute bout of continuous treadmill running in wild type (WT) and AMPK transgenic mice with double knock-in (DKI) mutations in the β subunit carbohydrate binding module that mediates glycogen binding. **Results**: Over 200 total metabolites were identified/annotated across liver and skeletal muscle, including 45 metabolites responsive to exercise (*p* < 0.05; FDR < 0.1). Exercise-regulated metabolites included known metabolic pathways and metabolites never associated or with only emerging evidence related to exercise (e.g., ergothioneine) and/or AMPK-glycogen interactions (N6,N6,N6-trimethyl-L-lysine, a precursor of L-carnitine). **Conclusions**: Liver and skeletal muscle metabolomic profiles displayed shifts between WT and DKI mice at rest, with shifts also detected following a continuous acute exercise bout. An interaction effect was also observed in skeletal muscle, suggesting differential muscle metabolite responses to acute exercise in DKI mice that may contribute to their functional impairments in metabolic control and exercise capacity versus WT. Collectively, these findings expand the molecular landscape of acute exercise and reveal liver and muscle metabolites underlying exercise-induced metabolic responses.

## 1. Introduction

Exercise represents a major challenge to energy homeostasis, with adenosine triphosphate (ATP) turnover increasing up to 100-fold within cells in response to exercise [1]. One of the key regulators of cellular energy homeostasis is the AMP-activated protein kinase (AMPK), an αβγ heterotrimeric enzyme able to directly sense cellular energy levels by competitively binding ATP, adenosine diphosphate (ADP) and adenosine monophosphate (AMP) [2]. During exercise, increased cellular levels of ADP and AMP relative to ATP lead to AMPK activation. Once activated, AMPK inhibits anabolic processes such as protein and lipid synthesis while concurrently activating catabolic pathways including glycolysis, lipolysis, and protein breakdown to maintain energy homeostasis [2].

AMPK also senses stored energy in the form of glycogen—an important energy reserve in liver and skeletal muscle utilized as the preferred fuel source during moderate- and high-intensity (>70% of VO_2max_) exercise [3]. AMPK-glycogen binding is mediated by the carbohydrate binding module (CBM) within the AMPK β1 and β2 regulatory subunit isoforms [4,5]. In rodents, the β1 and β2 isoforms are predominantly expressed in liver and skeletal muscle, respectively [6]. Specific tryptophan residues within the CBM (β1 W100 and β2 W98) are crucial for AMPK-glycogen binding in vitro [4,5]. Indeed, a growing body of evidence has highlighted interactive roles of AMPK and glycogen at the molecular, cellular and physiological levels [6]. Further research into these AMPK-glycogen interactions in vivo and their precise physiological roles in metabolic homeostasis and exercise is therefore warranted.

Previous in vivo mouse studies from our group have shown that whole-body knock-in mutation of these W100 and W98 amino acid residues to alanine (A) within AMPK’s CBM disrupts metabolic homeostasis and exercise capacity [7,8,9]. Mutations in either the β2 isoform CBM or both β1/β2 isoforms’ CBM were associated with increased fat mass, impaired glucose handling, increased reliance on carbohydrate utilization as fuel and decreased maximal exercise capacity relative to wild type (WT) mice [7,8,9]. Given the metabolome is considered as a real-time snapshot of the cumulative changes occurring in all upstream biological layers and their interactions with the environment [10], we recently used an untargeted metabolomics approach to determine the molecular responses to acute exercise and the effects of AMPK-glycogen binding disruption in mouse plasma [11]. Mice with AMPK β1 (W100A)/β2 (W98A) double knock-in (DKI) mutations to chronically disrupt whole-body AMPK-glycogen interactions had significantly different overall plasma metabolomic profiles at rest relative to WT mice, but these metabolomic profiles converged following acute exercise. AMPK DKI mice showed increased plasma amino acid levels relative to WT mice in the resting state but decreased amino acid levels following exercise. This interaction effect suggested an increased reliance on amino acid metabolism underlying the response to exercise in DKI versus WT mice.

However, insight into the molecular and metabolic mechanisms regulating responses to both exercise and AMPK-glycogen binding disruption captured by analyzing the plasma metabolome in isolation is limited. Therefore, in the present study we hypothesized that substantial alterations in the metabolomic profiles of the primary glycogen-storage tissues, liver and skeletal muscle, would be sustained as a result of acute exercise and AMPK DKI mutations. The primary outcome of this study was to characterize liver and skeletal muscle metabolic responses to an acute bout of continuous treadmill running, as well as the effects of disrupting AMPK-glycogen interactions at rest and following exercise. To elucidate whether the genotype differences in fuel utilization and maximal exercise capacity previously observed were associated with altered mitochondrial function and contributed to differences in metabolomic profiles between DKI and WT mice, we also performed mitochondrial bioenergetics in red and white gastrocnemius muscle collected in both genotypes.

## 2. Materials and Methods

### 2.1. Mouse Model

We utilized the AMPK DKI mouse model generated by our group [8] and described in detail previously [11]. The Mouse Engineering Garvan/ABR (MEGA) Facility (Moss Vale, NSW, Australia) used CRISPR/Cas9 gene editing on a C57BL/6J background to generate whole-body AMPK Prkab1 W100A (β1 W100A) and Prkab2 W98A (β2 W98A) single KI mice [7,12]. Homozygous single KI mice were subsequently crossbred to generate Prkab1 W100A/Prkab2 W98A DKI mice and were compared to age-matched WT control mice. Confirmatory genotyping was performed by TransnetYX (Cordova, TN, USA) using tail samples.

All tissue samples used for metabolomic analyses and all anthropometric and physiological data were collected from the exact same mice utilized in plasma metabolomics experiments (*n* = 44 total). Anthropometric and physiological characteristics were previously reported with plasma metabolomics data in [11]. In summary, AMPK DKI mice displayed ~10% increase in total body mass, with ~65% increase in whole-body fat mass but no change in lean mass relative to WT mice. Maximal running speed in AMPK DKI mice was also decreased ~20% relative to WT mice. An additional cohort of WT and AMPK DKI mice (*n* = 2 and *n* = 4, respectively) were utilized for mitochondrial respiration assays, with 50 mice total utilized across the study. All mouse procedures were performed under the approval of the St. Vincent’s Hospital (Melbourne, VIC, Australia) Animal Ethics Committee (approval numbers 025-15 and 011-19), in accordance with all NHMRC requirements and conforming to National Institutes of Health animal research guidelines (NIH Publications No. 8023, revised 1978) and the Australian code for the care and use of animals for scientific purposes (8th Edition 2013; updated 2021). Mice were kept in pathogen-free microisolator cages (2–5 mice per cage) on standard 12:12 h dark-light cycles with controlled temperature (21 °C), humidity and bedding. Mice were allowed ad libitum water access and standard chow diet (29% starch 20% protein and 6% fat, Barastoc, Ridley Agriproducts, Pakenham, VIC, Australia).

### 2.2. Metabolomic Analysis

#### 2.2.1. Continuous Acute Exercise Bout and Tissue Collection

Male DKI and WT mice aged 12–16 weeks were acclimatized to treadmill running (Exer 3/6, Columbus Instruments, Columbus, OH, USA) for four consecutive days at progressively increasing speeds prior to performing a maximal running speed test. Full details of the treadmill acclimatization and maximal running speed test protocols are provided in [11]. Mice were then randomly assigned to either an exercise or rested control group. Following 2–3 days of recovery, mice assigned to the exercise group completed a 30 min-run at 70% of their individual maximal speed at a 0° incline, while rested control mice remained in their home cage. Upon completion of the exercise bout, both rested and exercised mice were immediately placed in a CO_2_ chamber for ~10 s prior to cardiac puncture and tissue collection. Euthanasia and sample collection were performed in the fed state (without standardization of last meal timing), between 0800 and 1000 h, corresponding to the start of the light cycle. Liver (with consistent liver lobes collected between mice) and gastrocnemius skeletal muscle samples were immediately snap frozen in liquid nitrogen and stored at −80 °C until further analysis.

#### 2.2.2. Sample Preparation

Tissue sample preparation and Liquid Chromatography–Mass Spectrometry (LC-MS) analyses were performed at Edith Cowan University (Joondalup, WA, Australia) according to previously described methods [13,14]. An average of 50 mg (wet weight) of snap-frozen liver and mixed gastrocnemius muscle from each mouse was chipped and freeze-dried overnight. Approximately 10 mg of tissue was then manually ground into fine powder with striated forceps on a glass dish ahead of extraction for LC-MS analysis. Extraction solution was prepared using methanol: acetonitrile: water (2/2/1 (*v*/*v*)) with isotopically labeled internal standards (valine-d8, phenylalanine-d8, caffeine-13C3, creatinine-d3, leucine-d3, sphingosine-d9, tryptophan-d5, trimethylamine N-oxide-d8, taurodeoxycholic acid-d5) at 1 ppm. Approximately 800 μL of extraction solution was added to each tissue sample (volume was corrected to the mass of each powdered tissue sample) and blank tube. Each tube was then vortexed for 10 s and placed on thermomixer agitator at 1200 rpm for 2 min at 4 °C and centrifuged (Heraeus Megafuge 8R, Thermo Fisher Scientific, Scoresby, VIC, Australia) for 10 min at 14,000 rpm. Following agitation, 100 µL of supernatant was added to two separate glass inserts in injection vials (one set of vials for each assay, detailed below). An additional 40 µL of remaining supernatant from each sample was combined to create a pooled quality control (QC) stock solution, which was then aliquoted into 20 LC-MS vials of 60 µL each. The sample injection sequence (block randomized by group [block size = 4]; WT-Rest, WT-Ex, DKI-Rest, DKI-Ex) was identical to that of our mouse plasma metabolomic study [11]. Block randomization was performed to ensure a balanced representation of genotypes and conditions across each block. The block-randomized sequence was generated using Microsoft Excel (version 2019), and the investigator conducting downstream analyses was blinded to group allocation. LC-MS system suitability was checked using the quality assurance methods described previously [15] and 8 pooled QC samples were injected at the beginning of the sequence to condition the column. Pooled QC samples were then injected following each block of experimental samples for assessment of analytical precision as reported previously [15].

#### 2.2.3. LC-MS

##### C18 Assay

All tissue samples were analyzed using an Ultra High-Pressure Liquid Chromatography pump Dionex UltiMate 3000 RScoupled to an Orbitrap Q-Exactive MS (Thermo Scientific, San Jose, CA, USA) fitted with a heated electrospray ionization (HESI) probe. Metabolite separation was performed using a reversed phase Hypersil GOLD column (100 mm × 2.1 mm, 1.9 μm particle size; Thermo Scientific, San Jose, CA, USA) with an in-line filter. Tissue samples were analyzed in positive and negative ionization modes using 0.1% formic acid in LC-MS water (solvent A) and 0.1% formic acid in LC-MS grade acetonitrile (solvent B). The following elution gradient was utilized: isocratic at 99% solvent A for 1 min, followed by an increase to 50% solvent B (1–2 min), then a linear increase to 99% solvent B over 7 min, which was maintained at 99% solvent B for 2 min. Initial conditions were returned over 2 min and held at 100% solvent A for equilibration (3 min). The flow rate was 0.3 mL/min for positive and negative modes, the injection volume was 4 μL, and the autosampler tray was set to 6 °C and column oven temperature to 45 °C. Full scans with data-dependent MS/MS were acquired with the Orbitrap mass analyzer. Full scans were acquired with a resolution of 70,000 at mass-to-charge ratio (*m*/*z*) 200 over the *m*/*z* range 70–1000 with the following electrospray ionization (ESI) conditions: source heater = 413 °C (positive mode) 350 °C (negative mode), sheath gas = 50 (positive mode) 35 (negative mode) (arbitrary units), auxiliary gas = 10 (arbitrary units), capillary temperature = 320 °C (positive mode) 350 °C (negative mode), ion spray voltage = 3.5 kV (positive mode) and 2.5 kV (negative ion mode), S-lens 50%, and automatic gain control = 1 × 10^−6^. MS/MS was performed at a resolution of 17,500 at *m*/*z* 200 for each sample with the higher energy collisional dissociation energy of 20 eV. Data acquisition was performed with Xcalibur software (v4.3, Thermo Scientific, San Jose, CA, USA). Prior to analysis, external calibration of the Orbitrap was carried out using ready-made calibration solutions (ESI-negative ion calibration and ESI-positive ion calibration solutions) purchased from Thermo Fisher Scientific (San Jose, CA, USA).

##### HILIC Assay

Tissue samples were analyzed using an Ultra High-Pressure Liquid Chromatography pump Dionex UltiMate 3000 RS coupled to an Orbitrap Q-Exactive MS (Thermo Scientific, San Jose, CA, USA) fitted with a HESI probe (positive ionization mode). Metabolite separation was performed on an Acquity UPLC Amide Column (100 mm × 2.1 mm, 1.7 μm particle size; Waters, Milford, MA, USA). Sample analysis was performed using LC-MS water buffered with 10 mM ammonium formate and 50 mM formic acid for a pH of 3 (solvent A) and 90:10 LC-MS acetonitrile and LC-MS grade water buffered with 10 mM ammonium formate and 50 mM formic acid (solvent B). The following elution gradient was used: isocratic at 100% solvent B for 1 min, followed by a linear increase to 40% solvent A (1–8 min), which was maintained at 40% solvent A for 1 min. Initial conditions were returned over 1 min, then held at 100% solvent B for equilibration (5 min). The flow rate was 0.4 mL/min. Injection volume was 4 μL, and the autosampler tray was set to 6 °C and column oven temperature to 45 °C. Full scans with data-dependent MS/MS were acquired with the Orbitrap mass analyzer. Full scan acquisition was performed at a resolution of 70,000 at mass-to-charge ratio (*m*/*z*) 200 over the *m*/*z* range 70–1000 with the following ESI conditions: source heater = 350 °C, sheath gas = 35 (arbitrary units), auxiliary gas = 10 (arbitrary units), capillary temperature 350 °C, ion spray voltage = 3.5 kV, S-lens 50%, and automatic gain control = 1 × 10^−6^. MS/MS was performed at a resolution of 17,500 at *m*/*z* 200 for each sample with the higher energy collisional dissociation energy of 20 eV. Data acquisition was performed with Xcalibur software (v4.3, Thermo Scientific, San Jose, CA, USA). Prior to analysis, external calibration of the Orbitrap was carried out using ready-made calibration solutions purchased from Thermo Fisher Scientific (San Jose, CA, USA).

#### 2.2.4. Data Processing and Metabolite Identification/Annotation

Following data acquisition, spectral processing was performed (i.e., HILIC POS and C18 POS and C18 NEG) in Compound Discoverer™ 3.1 (Thermo Scientific, San Jose, CA, USA) using an untargeted metabolomics workflow. Compound Discoverer™ version 3.1 was used for total ion chromatograms alignment along retention times (RT) based on an adaptive curve, with a 5 ppm mass tolerance and a maximum shift of 0.5 min. Detected features with a signal-to-noise ratio >5 and an intensity ≥1,000,000 in each dataset were merged into compounds according to ion adducts. Ions detected within the blanks were subtracted from the samples using the “mark background compounds” node. Online databases such as the human metabolome database: https://www.hmdb.ca (accessed on 9 March 2026), mzCloud: https://www.mzcloud.org (accessed on 9 March 2026), and the Kyoto Encyclopedia of Genes and Genomes: https://www.genome.jp/kegg/ (accessed on 9 March 2026)) were searched to putatively annotate metabolites. Metabolite identification was also performed by matching retention time and mass spectral fragmentation patterns to an in-house chemical standards library consisting of ~400 metabolites at the time of identification/annotation. All identifications and annotations were assigned in accordance with the Metabolomics Standards Initiative (MSI) levels [16]. Quality control-regularized spline correction (QC-RSC) was used to correct analytical signal drift [17,18]. Following standard protocols [15], metabolites with relative standard deviation (RSD) QC >20% or a Dispersion-ratio (D-ratio) >30% were removed from further statistical analyses based on their failure to meet acceptable measurement precision.

### 2.3. Mitochondrial Respiration in Skeletal Muscle Fibers

Male WT and DKI mice aged ~15–23 weeks were culled by cervical dislocation in the fed state between 0800 h and 0900 h. Gastrocnemius muscles were collected and placed in ice-cold BIOPS solution (50 mM MES, 7.23 mM K_2_EGTA, 2.77 mM CaK_2_EGTA, 20 mM imidazole, 0.5 mM DTT, 20 mM taurine, 5.77 mM ATP, 15 mM PCr, and 6.56 mM MgCl_2_·H_2_O; pH 7.1). Each muscle sample was trimmed of connective tissue and fat and divided into red and white muscle portions. Several small muscle bundles (~2.0–5.0 mg wet weight) were prepared by separating muscle along the longitudinal axis using fine-tipped forceps under a dissecting microscope. Fiber bundles were then treated with 40 µg/mL saponin for 30 min at 4 °C and subsequently washed in MiR05 respiration buffer (0.5 mM EGTA, 10 mM KH_2_PO_4_, 110 mM sucrose, and 1 mg/mL fatty acid-free BSA; pH 7.1) for 15 min as described previously Perry et al. (2012) [19].

Oxygen (O_2_) consumption measurements from permeabilized muscle fibers were performed in MiR05 respiration medium using an Oxygraph-2K (Oroboros Instruments, Innsbruck, Austria) at 37 °C in the presence of 25 µM blebbistatin [20]. ADP-stimulated respiration was determined from fibers prepared from red and white muscle. Titrations were initiated in the presence of 10 mM pyruvate and 5 mM malate and ADP was titrated at various concentrations. Glutamate (10 mM) and succinate (10 mM) were added to quantify maximum mitochondrial respiration. Finally, outer mitochondrial membrane integrity was determined by adding 10 µM cytochrome c, with <10% increase in respiration indicating outer mitochondrial membrane integrity. Experiments were performed in duplicate and data were normalized to muscle fiber bundle wet weight. The apparent K_m_ for ADP was determined as previously described [19].

### 2.4. Statistical Analysis

Statistical analysis of metabolomics datasets was performed using a two-way ANOVA to test the null hypothesis that there were no differences between rested/exercised conditions and/or WT/DKI genotypes for each individual metabolite. False discovery rate (FDR) estimation to account for multiple comparisons was performed using the Storey FDR method [21], and *p* values < 0.05 with a FDR < 0.1 were considered statistically significant. Agglomerative assessment of similarities between metabolite concentrations across the complete dataset was performed by hierarchical cluster analysis (HCA) using Pearson’s correlation coefficient as the similarity measure, and linkage performed using Ward’s method [22]. The lowest linkages (i.e., closest metabolite clusters) within the HCA dendrogram indicate metabolites that display the most similar responses. All metabolites were then combined into a single data matrix and applied to principal component–canonical variate analysis (PC-CVA) [23] to visualize the multivariate discrimination between the four experimental groups. To avoid overfitting, 5-fold cross validation was used to choose the optimal number of principal components to apply to the CVA model. 95% CI for the model coefficients were calculated using bootstrap resampling [24]. Prior to the above statistical analyses, metabolite concentrations were log_10_ transformed and scaled to unit variance (autoscaled). Metabolomics statistical analysis was performed using MATLAB^®^ version R2023b (MathWorks, Natick, MA, USA). Oroboros O2k experiments were analyzed statistically using Student’s *t*-test or one- or two-way ANOVA with Fisher’s least significant difference as post hoc analysis where applicable. These statistical analyses were performed using GraphPad Prism software version 8.4.3 (GraphPad Software, San Diego, CA, USA).

## 3. Results

As detailed in the Section 2, anthropometric and physiological characteristics of the exact WT and DKI mice utilized in the present study, including total body mass, fat mass and lean mass, as well as exercise capacity (i.e., maximal running speed) were published with our group’s plasma metabolomics data in [11].

### 3.1. Metabolomic Analyses Reveal Mouse Liver Metabolites Associated with Continuous Acute Exercise and Disruption of AMPK-Glycogen Binding

Using untargeted metabolomics and following the standard reporting guidelines from the MSI [16], a total of 150 liver metabolites were identified/annotated. Analysis via two-way ANOVA revealed 29 metabolites with significant differences between conditions (rested versus exercised, *p* < 0.05; FDR < 0.1), whereas no significant differences were observed between genotypes (WT versus DKI). All identified/annotated liver metabolites, their molecular characteristics and respective statistical relationships are listed in Supplementary Table S1.

#### 3.1.1. Correlated Metabolite Clusters

**Figure 1:**
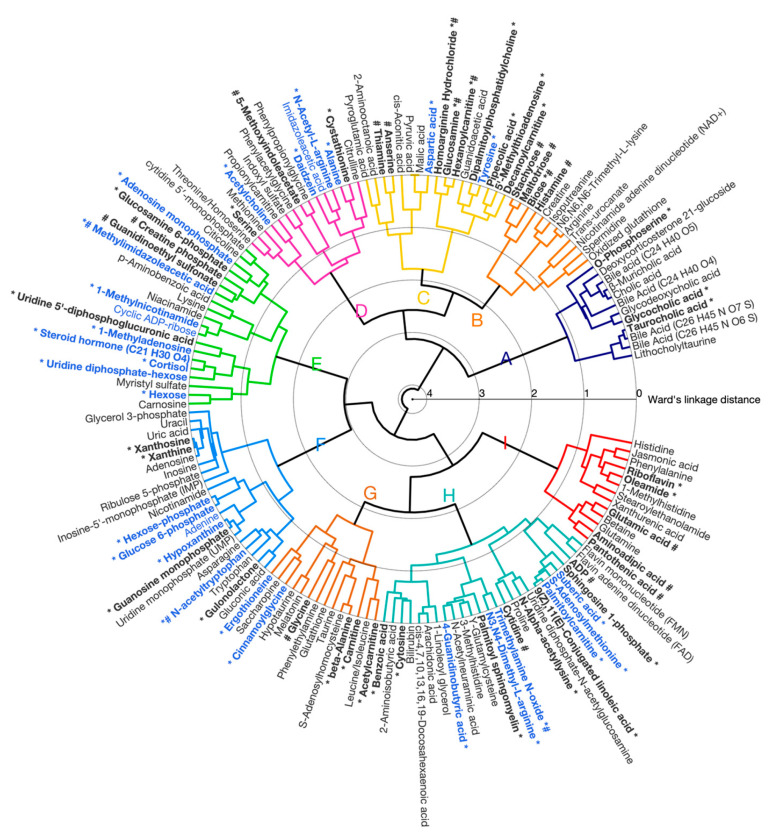
Hierarchical cluster analysis (HCA) dendrogram of identified/annotated mouse liver metabolites. Agglomerative clustering of individual metabolites based on pairwise correlation is shown. The lowest linkages within the HCA dendrogram (linkage distances ranging from 0 to 4 using Ward’s method) indicate metabolites that display similar relative responses between the experimental groups. Nine clusters were observed (labeled A–I). Metabolite labels are colored to reflect the results of two-way ANOVA after filtering using a false discovery rate (FDR) of 0.1 (blue = significant effect of condition; black = no significance or FDR > 0.1). *: Metabolites that significantly (*p* < 0.05) contributed to the model along canonical variate 1 (CV1, Figure 2); #: Metabolites that significantly (*p* < 0.05) contributed to the model along CV2.

Hierarchical clustering of these 150 liver metabolites resulted in nine clusters reflecting differences in patterns of metabolite abundance across experimental groups (WT-Rest, WT-Ex, DKI-Rest and DKI-Ex) based on pairwise Pearson’s correlation similarity. The results are displayed as a circular dendrogram (Figure 1), and averaged z-score metabolite responses for each cluster are shown in Supplementary Figure S1. No significant differences in the average response for metabolites in Cluster A (12 metabolites, predominantly bile acids) were observed between genotypes or conditions. Cluster B (12 metabolites, including carbohydrate species, creatine and histamine) showed a significant genotype effect (*p* < 0.04) with higher average metabolite abundance in DKI relative to WT liver. Cluster C (17 metabolites, composed of various metabolite species including amino acids, tricarboxylic acid [TCA] cycle intermediates, acylcarnitines), did not show statistically significant differences between genotypes in liver. In contrast, Cluster D (15 metabolites, comprising acetylcholine, amino acids and derivatives) was significantly decreased in the exercise condition (*p* < 0.01), regardless of genotype. Cluster E (20 metabolites, including creatine phosphate, adenosine monophosphate [AMP], cortisol, carnosine) showed both an exercise- and genotype-association (*p* = 1.13 × 10^−5^ and *p* = 0.01, respectively), while no significant condition or genotype associations were observed for Clusters F (21 metabolites such as glucose-6-phosphate, xanthine and hypoxanthine) and G (14 metabolites including carnitine, acetylcarnitine and amino acids). Finally, Clusters H (26 metabolites, including various species such as cofactors, complex lipids and amino acid derivatives) and I (13 metabolites, including vitamins and amino acids) did not show statistically significant differences between conditions and genotypes.

**Figure 2:**
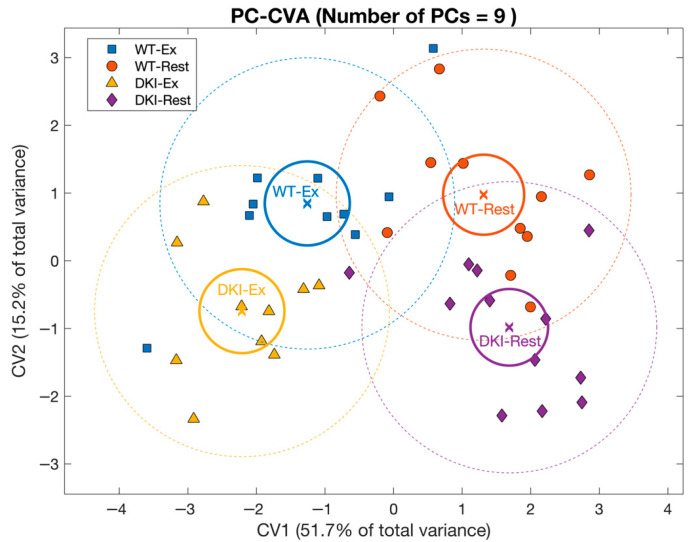
Principal component–canonical variate analysis (PC-CVA) showing overall mouse liver metabolite profile differences between genotypes at rest and after exercise. Scores plot of canonical variate 1 (CV1) versus canonical variate 2 (CV2). Each point (circle, square, diamond or triangle) represents a single sample [WT-Rest (*n* = 11), WT-Ex (*n* = 10), DKI-Rest (*n* = 12), DKI-Ex (*n* = 10)]. The mean (x) of each group is surrounded by a 95% confidence interval of the mean (full-line circles) and 95% confidence interval of membership in each sample group (dashed-line circles). Sample group means are considered significantly different when the 95% confidence interval of the means do not overlap.

#### 3.1.2. Principal Component–Canonical Variate Analysis

Between-group multivariate differences in liver metabolite profiles with respect to condition and genotype were next determined by performing PC-CVA (Figure 2). Canonical variate (CV1) explained >50% of the total variance in the overall liver metabolomic dataset, whereas CV2 explained ~15% of the total variance. CV1 demonstrated significant differences in the multivariate mean between the rested and exercised conditions for both WT and DKI, while CV2 showed significant differences between WT and DKI mouse liver metabolomes at rest and following exercise. The contribution of each metabolite (with 95% confidence intervals) to CV1 and CV2 are presented in Supplementary Figure S2 (with significant metabolites indicated in red).

**Figure 3:**
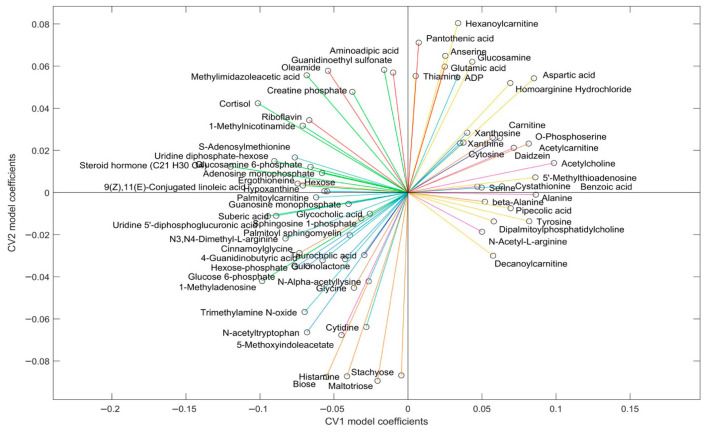
Loading plot showing the influence (model coefficient value) of each metabolite that significantly (*p* < 0.05) contributed to the separation observed in the scores plot (Figure 2). The direction of the coefficient vector maps directly to the direction of the data points in the scores plot relative to the origin.

The liver metabolites significantly contributing to the model are depicted in Figure 3. In this plot, the position of each metabolite relative to the origin indicates the directionality of sample groups in the scores plot (Figure 2). Metabolite grouping along the x-axis are associated with the exercise intervention, meaning that metabolites on the left and right are increased and decreased with exercise, respectively. Metabolites along the y-axis are indicative of genotypic differences, with metabolites in the upper quadrants being increased in WT relative to DKI and metabolites in the lower quadrants being increased in DKI relative to WT liver. Cluster groups in Figure 1 and Figure 3 are color-matched and the significant CV coefficients are labeled in the HCA plot.

### 3.2. Metabolomic Analyses Reveal Skeletal Muscle Metabolites Associated with Continuous Acute Exercise and Disrupting AMPK-Glycogen Binding

In accordance with the standard reporting guidelines from the MSI [16], 92 gastrocnemius muscle metabolites were identified/annotated using untargeted metabolomics. Analysis via two-way ANOVA demonstrated 16 metabolites with significant differences (*p* < 0.05; FDR < 0.1) between conditions (rested versus exercised), whereas only one metabolite, N6,N6,N6-trimethyl-L-lysine, was significantly different between DKI and WT muscle (*p* = 4.82 × 10^−4^; FDR < 0.04). Identified/annotated muscle metabolites, their molecular characteristics and respective statistical relationships are listed in Supplementary Table S2.

#### 3.2.1. Correlated Metabolite Clusters

**Figure 4:**
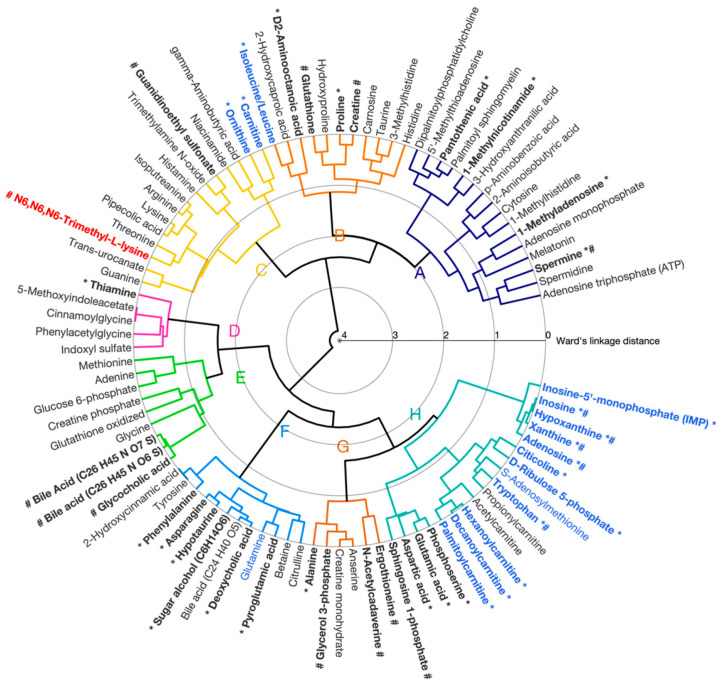
Hierarchical cluster analysis (HCA) dendrogram of identified/annotated mouse gastrocnemius muscle metabolites. Agglomerative clustering of individual metabolites based on pairwise correlation is shown. The lowest linkages within the HCA dendrogram (linkage distances ranging from 0 to 4 using Ward’s method) indicate metabolites that display similar relative responses between the experimental groups. Eight clusters were observed (labeled A–H). Metabolite labels are colored to reflect the results of the two-way ANOVA after filtering using a false discovery rate (FDR) of 0.1 (blue = significant effect of condition; red = significant genotype effect; black = no significance or FDR > 0.1). *: Metabolites that significantly (*p* < 0.05) contributed to the model along canonical variate 1 (CV1, Figure 5); #: Metabolites that significantly (*p* < 0.05) contributed to the model along CV2.

A total of eight metabolite clusters were identified from the hierarchical clustering of these 92 identified/annotated muscle metabolites. The circular dendrogram and averaged z-score metabolite responses for each cluster are shown in Figure 4 and Supplementary Figure S3, respectively. No significant differences were observed in Cluster A (16 metabolites, including ATP, AMP and melatonin) between genotypes or conditions. Cluster B (11 metabolites, primarily amino acids and derivatives) was significantly increased in DKI relative to WT muscle (*p* < 0.01). No significant associations with either genotype or condition were observed in Clusters C (15 metabolites, mainly composed of carnitine, ornithine and several amino acids), D (5 metabolites, including thiamine and glycine derivatives), E (9 metabolites, including bile acids, oxidized glutathione and glucose-6-phosphate) and F (12 metabolites including deoxycholic acid, hypotaurine and citrulline). In contrast, Cluster G (6 metabolites including alanine, glycerol-3-phosphate and creatine monohydrate) showed a significant interaction effect between genotype and condition (*p* < 0.05). Specifically, metabolites in Cluster G displayed significantly higher abundance in WT versus DKI gastrocnemius muscle at rest and were decreased following exercise in WT muscle but increased following exercise in DKI muscle. Finally, Cluster H (18 metabolites, including various several carnitine species, adenosine, inosine and hypoxanthine) was significantly increased in the exercised condition (*p* = 3.58 ×10^−4^), independent of genotype.

**Figure 5:**
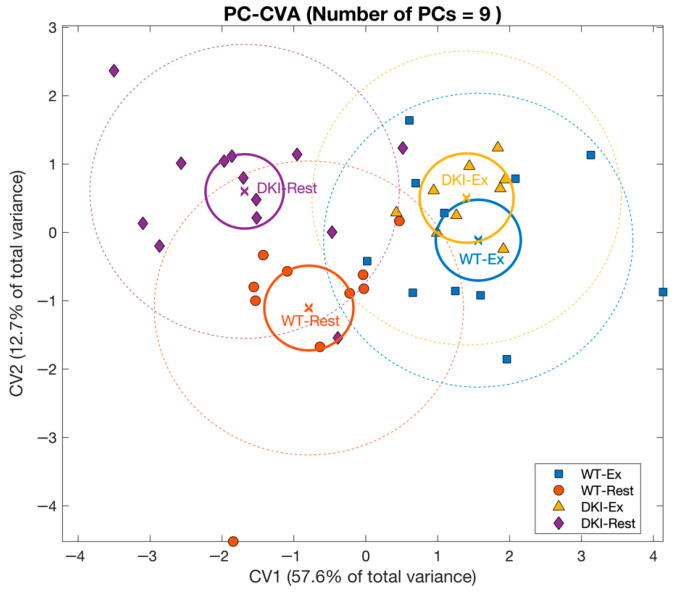
Principal component–canonical variate analysis (PC-CVA) showing overall mouse gastrocnemius muscle metabolite profile differences between genotypes at rest, and between conditions (exercise versus rest). Scores plot of canonical variate 1 (CV1) versus canonical variate 2 (CV2). Each point (circle, square or triangle) represents a single sample [WT-Rest (*n* = 10), WT-Ex (*n* = 11), DKI-Rest (*n* = 13), DKI-Ex (*n* = 10)]. The mean (x) of each group is surrounded by a 95% confidence interval of the mean (full-line circles) and 95% confidence interval of membership in each sample group (dashed-line circles). Sample group means are considered significantly different when the 95% confidence interval of the means do not overlap.

#### 3.2.2. Principal Component–Canonical Variate Analysis

PC-CVA was next applied to the muscle metabolomic dataset to identify between-group multivariate differences in overall metabolite profiles with respect to condition and genotype (Figure 5). Canonical variate (CV1) explained ~58% of the total variance in the overall muscle dataset, whereas CV2 explained ~13% of the total variance. CV1 demonstrated significant muscle metabolomic differences in the multivariate mean between the rested and exercised conditions for both WT and DKI muscle, while CV2 showed significant differences between WT and DKI muscle metabolomes at rest, but not following exercise. The contribution of each metabolite (with 95% confidence intervals) to CV1 and CV2 is presented in Supplementary Figure S4 (with significant metabolites indicated in red).

**Figure 6:**
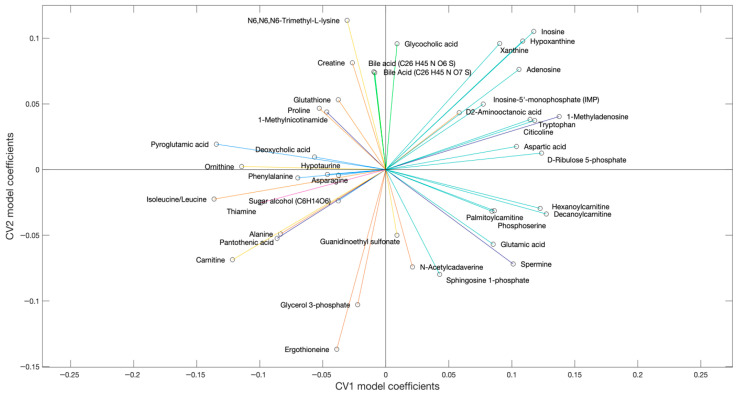
Loading plot showing the influence (model coefficient value) of each metabolite that significantly (*p* < 0.05) contributed to the separation observed in the scores plot (Figure 5). The direction of the coefficient vector maps directly to the direction of the data points in the scores plot relative to the origin.

The muscle metabolites that contribute significantly to the model are depicted in Figure 6. The position of each metabolite relative to the origin indicates the directionality of sample groups in the scores plot (Figure 5). In this muscle dataset, metabolite grouping along the x-axis represent differences associated with the exercise intervention and metabolites on the right depict increased abundances in the exercised relative to rested muscle. Along the y-axis, metabolites demonstrate genotypic differences, with increased abundances in DKI relative to WT muscle when metabolites are positioned in the upper part of the figure. Cluster groups in Figure 4 and Figure 6 are color-matched and the significant CV coefficients are labeled in the HCA plot.

### 3.3. Mitochondrial Respiration Is Not Different Between WT and DKI Mouse Permeabilized Red and White Gastrocnemius Muscle Fibers

**Figure 7:**
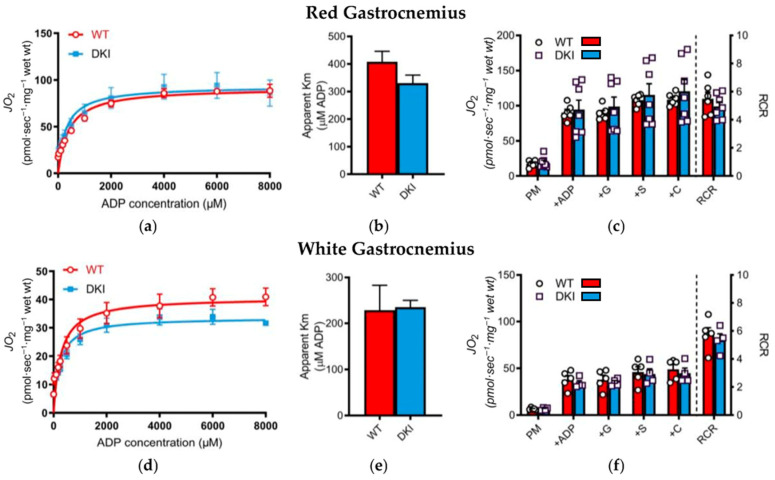
Mitochondrial bioenergetics was assessed in permeabilized fibers prepared from red (**a**–**c**) and white (**d**–**f**) gastrocnemius muscle from WT and DKI mice. Michaelis–Menten kinetic curves were generated to assess ADP respiratory kinetics (**a**,**d**) in the presence of pyruvate and malate in the respiration media to calculate the apparent Km (**b**,**e**). Respiration was determined in the absence (state 4) and presence (state 3) of ADP, supported by complex I (pyruvate, malate and glutamate) and complex II (succinate) substrates (**c**,**f**). P: pyruvate; M: malate; G: glutamate; S: succinate; C: cytochrome c; RCR: respiratory control ratio (state 3/state 4). Values are expressed as means ± SEM. *n* = 5–7 permeabilized fibers per genotype and muscle type (i.e., red and white gastrocnemius).

Metabolites in Cluster G displayed opposite responses following exercise between genotypes, with glycerol-3-phosphate being the predominant driver of these overall metabolite profile changes within Cluster G. Glycerol-3-phosphate is an intermediate metabolite involved in several energy metabolism pathways including glycolysis, gluconeogenesis, and glycerolipid (e.g., triacylglycerol) and fatty acid utilization [25]. In contrast, there were no genotypic differences observed in Cluster A which included both ATP and AMP, while Cluster H, containing multiple carnitine species indicative of fatty acid flux, increased to a similar extent post exercise in both WT and DKI animals. We have previously shown that DKI mice display lower rates of whole-body fat oxidation and/or greater rates of carbohydrate oxidation, despite having similar mitochondrial content and substrate transporter protein levels (i.e., CPT1b and GLUT4) compared to WT [8,9]. Therefore, to determine whether alterations in mitochondrial function contributed to the altered metabolic profile and decreased maximal exercise capacity of DKI mice [7,8,9], we examined mitochondrial bioenergetics in red and white gastrocnemius muscle collected in both genotypes. There were no genotypic differences detected in ADP respiratory kinetics when submaximal boluses of ADP were titrated in either red (Figure 7a) or white (Figure 7d) gastrocnemius muscle, nor were there any differences in ADP sensitivity as assessed by the apparent Km (Figure 7b,e). Overall mitochondrial respiration (Figure 7c,f) was also similar between genotypes.

## 4. Discussion

Research from our group over the past five years has uncovered the phenotypic consequences of mutating key tryptophan residues in AMPK’s CBM (i.e., β1 W100 and β2 W98) in vivo, using transgenic mouse models with whole-body AMPK β isoform-specific KI and DKI mutations [7,8,9]. However, the molecular mechanisms underlying the whole-body phenotypic differences in exercise capacity and metabolic control (e.g., reduced maximal running speed, increased adiposity, greater whole-body reliance on carbohydrate utilization and/or reduced fat oxidation relative to WT) associated with the AMPK β DKI mutation remain unclear. It should also be acknowledged that while AMPK-glycogen binding is well-established in vitro, no in vivo models have directly demonstrated AMPK-glycogen binding [26].

Nonetheless, the phenotypes resulting from the amino acid mutations in AMPK’s β1 and β2 CBM presented here and in previous work from our group [7,8,9,10] highlights the complexity of AMPK and its numerous interactions, as well as the need to better understand the physiological roles of AMPK and its CBM. To address some of these knowledge gaps and build on our plasma metabolomic findings [11], we performed untargeted metabolomic analysis of the primary glycogen-storing tissues liver and skeletal muscle collected from the same mice as the previous plasma metabolomics analyses [11].

In liver, metabolomic analyses revealed significant mean differences in overall metabolite profiles between WT and DKI mice, and between rested and exercised conditions (Figure 2). Unlike univariate analyses that only detect individual mean metabolite differences between experimental groups in an independent manner, multivariate analyses such as PC-CVA test all metabolites simultaneously. This approach captures joint patterns/covariance in metabolite networks, making it more sensitive to small but consistent changes across many related metabolites [27]. Three metabolite clusters were associated with the DKI genotype and/or exercise condition (Supplementary Figure S1). Cluster B metabolites, including histamine and several carbohydrate species (MSI level 1), showed increased mean abundance in DKI versus WT liver (*p* < 0.04) and were amongst the most potent drivers of overall metabolite profile genotypic differences (Figure 3). Histamine regulates numerous physiological and pathophysiological processes [28]. While increased skeletal muscle histamine is an essential physiological response to exercise training adaptations [29], increased hepatic levels of histamine have also been shown to be associated with the progression of pathophysiological conditions including liver disease [30]. Whether increased hepatic histamine levels observed in DKI mice are associated with increased inflammation and impaired liver health remains uncertain and outside the scope of the present study. However, this would be a plausible hypothesis given the close relationship between increased levels of adiposity and inflammation, including liver inflammation [31], potentially consistent with the observed increases in adiposity and liver fat content in isoform-specific AMPK β isoform-specific KI and DKI mice [7,8,11]. Interestingly, while other groups have reported exercise-induced remodeling of hepatic bile acids in mice [32,33], no significant effects of acute continuous exercise were found in the present study (Cluster A). However, several studies demonstrated exercise effects on mouse hepatic bile acid levels included chronic exercise protocols [32,33]. This potentially suggests that repeated exercise bouts are necessary to elicit significant remodeling of the liver bile acids identified/annotated in the present study.

Cluster D metabolites including alanine, cystathionine, serine (MSI level 1) and acetylcholine (MSI level 2) were on average significantly decreased with exercise (*p* < 0.01) as shown in Supplementary Figure S1. Recent work from Olsen et al. (2020) in male cyclists have demonstrated increased circulating levels of glutathione—a downstream product of cystathionine and a major systemic antioxidant—in response to exhaustive exercise [34]. Decreased hepatic levels of cystathionine and its component serine may therefore suggest enhanced hepatic production and release of glutathione into the bloodstream to help combat exercise-induced oxidative stress [35,36,37].

Cluster E metabolites were significantly increased in association with exercise (*p* = 1.13 × 10^−5^) and significantly reduced in DKI versus WT mouse liver (*p* < 0.02) as shown in Supplementary Figure S1). Metabolites such as AMP and cortisol (MSI level 1) contributed to the exercise-associated metabolite profile separation, while metabolites including methylimidazoleacetic acid (MSI level 1) and creatine phosphate (MSI level 2) contributed to genotype metabolite profile separation (Supplementary Figure S2 and Figure 3). During continuous acute exercise, mouse liver is placed under substantial energetic stress, rodents relying predominantly on blood borne rather than intramuscular substrates during exercise [38], with elevated AMP and cortisol levels as surrogates for increased exercise-induced ATP-turnover [39]. As a catabolic hormone, cortisol stimulates mobilization of energy stores by promoting lipolysis, gluconeogenesis and protein breakdown [40]. Although corticosterone is the main glucocorticoid hormone produced in rodents, cortisol can also be found in rodents at lower concentrations and can increase following exposure to various stress-inducing stimuli [41,42,43]. Methylimidazoleacetic acid is the main end-product of histamine catabolism [44]. Reduced methylimidazoleacetic acid levels in DKI liver may therefore suggest decreased levels of histamine catabolism, given the increased abundance of hepatic histamine observed in DKI versus WT liver. Creatine phosphate (PCr) is an essential component of mitochondrial function and the ATP-PCr system that serves as a source of phosphate for ATP resynthesis that can be rapidly mobilized during high ATP turnover (e.g., during short and intense/explosive exercise) and acts as a cellular energy buffer [45]. Of relevance, recent metabolomic analyses performed on white adipose tissue showed that perturbations in the phosphocreatine/creatine ratio in was associated with increased levels of adiposity in individuals with obesity [46]. This increased phosphocreatine/creatine ratio reportedly resulted in changes in the ATP/ADP ratio and attenuated AMPK activity [46]. In the present study, there was a significant contribution of putatively annotated PCr to genotypic differences (CV2) potentially indicating decreased PCr abundance in DKI versus WT liver. As no change in hepatic creatine (MSI level 1) was observed, this suggests a potential decrease in the DKI hepatic phosphocreatine/creatine ratio may also be associated with increased whole-body adiposity, as previously reported in DKI mice [8,11]. Nevertheless, further investigations are warranted to determine absolute tissue concentrations of creatine and PCr, and investigating different species and tissue types is warranted to better understand the relationship between phosphocreatine/creatine metabolism and AMPK activity.

In gastrocnemius muscle, univariate analyses revealed putatively annotated N6,N6,N6-trimethyl-L-lysine (MSI level 2) as the only metabolite significantly associated with DKI mutation while 16 metabolites were significantly associated with exercise (Supplementary Table S2). N6,N6,N6-trimethyl-L-lysine is an N-methylated derivative of L-lysine that serves both as a component of histone proteins with regulatory functions in epigenetic processes, and as a precursor of L-carnitine, a key component of fat oxidation [47,48]. Increased N6,N6,N6-trimethyl-L-lysine may indicate dysregulated protein methylation [47], with potential impact on fatty acid metabolism through decreased carnitine biosynthesis [49]. As displayed in Figure 6, multivariate analyses demonstrated a reduced carnitine abundance in DKI relative to WT gastrocnemius muscle. However, additional work is required to confirm this hypothesis. Significant mean differences in overall metabolite profiles were also observed between the rested and exercised conditions, and between genotypes at rest, but not following continuous acute exercise (Figure 5). These overall differences in metabolite profile in gastrocnemius muscle reveal similar patterns to those observed in our plasma metabolomic study, which demonstrated an interaction between condition and genotype and convergence of DKI and WT overall metabolite profiles following continuous acute exercise [11]. Similar to observations in the liver, three muscle metabolite clusters were associated with the DKI genotype and/or exercise condition.

Cluster B mean metabolite abundance was increased in DKI versus WT mouse muscle (*p* < 0.01, Supplementary Figure S3) with glutathione and creatine (MSI level 1) as the main drivers of genotypic differences (Supplementary Figure S4 and Figure 6). Increased intramuscular glutathione in DKI relative to WT mice may potentially indicate an adaptive response to increased levels of oxidative stress. In contrast to the observed decrease in putatively annotated PCr in DKI versus WT liver, no changes in intramuscular putatively annotated PCr (MSI level 2) levels were observed between conditions and genotypes. However, increased abundance in muscle creatine in DKI relative to WT contributed to genotype overall profile differences, suggesting potential changes in phosphocreatine/creatine ratio.

Cluster G displayed a significant interaction effect between genotype and condition (*p* < 0.05) with opposite mean metabolite abundance changes following exercise in DKI versus WT gastrocnemius muscle (Supplementary Figure S3). As outlined above, glycerol-3-phosphate (MSI level 1) was the predominant metabolite driving DKI versus WT overall metabolite profiles within Cluster G. These metabolites were decreased on average following continuous acute exercise in WT gastrocnemius muscle but increased in DKI muscle. Given the numerous energy metabolic pathways involving glycerol-3-phosphate [25], measuring additional sets of metabolites related to these pathways in future studies would help reveal the potential mechanism(s) underlying this metabolite’s observed interaction between genotypes and conditions.

Finally, Cluster H metabolite mean abundance was significantly increased in association with exercise (*p* = 3.58 × 10^−4^) with no observed genotypic differences (Supplementary Figure S3). From the 18 metabolites within cluster H, 14 significantly contributed to exercise-associated differences in overall metabolite profiles and supported univariate analyses (Figure 4 and Figure S4). Cluster H included long-, medium- and short-chain acylcarnitines, amino acids, as well as several metabolites of purine metabolism such as adenosine, inosine, xanthine, hypoxanthine (MSI level 1) and putatively annotated (MSI level 2) inosine monophosphate (IMP). Exercise-induced increases in purine metabolites are well-established responses to energy stress and biomarkers of increased ATP turnover. These metabolite responses to acute exercise in mouse skeletal muscle are supported by previous findings showing increased purine metabolites, as well as amino acids and TCA cycle intermediates in interstitial fluids of rat skeletal muscle following a single bout of exercise [50]. Likewise, increased intramuscular levels of fatty acylcarnitines in response to exercise are well documented and have been recently reviewed [10]. The skeletal muscle fatty acylcarnitine profiles observed in the present study suggest there are no differences in fat utilization in DKI versus WT skeletal muscle in response to acute submaximal exercise. This absence of genotype differences in intracellular lipid metabolites is consistent with previous work by our group, which did not detect any genotypic differences in: (1) whole-body fat oxidation rates assessed by indirect calorimetry during exercise [9]; (2) serum NEFA and plasma acylcarnitines [9,11]; and (3) the fat transport protein, carnitine palmitoyltransferase 1b (CPT1b) [9]. However, targeted analysis and/or greater coverage of metabolites involved in substrate utilization may help pinpoint potential metabolite differences underlying the observed increases in whole-body carbohydrate oxidation in DKI mice at rest and during continuous acute exercise [8,9].

While skeletal muscle ATP (putatively annotated, MSI level 2) and AMP (MSI level 1) levels were not altered by condition or genotype (Cluster A), a reduction in mitochondrial ADP sensitivity (i.e., greater ADP concentration to achieve a given absolute mitochondrial flux) may reflect greater metabolic stress and increased reliance on carbohydrate metabolism to support ATP production [51]. Indeed, ADP plays a central role in regulating the activation and subsequent flux through rate-limiting enzymes in carbohydrate metabolism. Furthermore, previous work has demonstrated that other rodent models of obesity/insulin resistance exhibit reduced mitochondrial respiration across a range of submaximal ADP concentrations [52,53]. Given we have previously demonstrated DKI mice share a similar phenotype with increased adiposity and glucose intolerance coupled with greater whole-body carbohydrate utilization at rest and during acute exercise [8,9], we utilized an ADP titration protocol to interrogate carbohydrate-supported mitochondrial ADP respiratory kinetics in both red and white gastrocnemius muscle. However, we were unable to detect any difference in assessments of mitochondrial bioenergetics including ADP sensitivity and mitochondrial capacity between genotypes. Mitochondrial content is also similar between DKI and WT mice [9], suggesting alterations in mitochondrial function cannot explain the reduction in exercise capacity and increased reliance on carbohydrate in DKI mice.

The present study also reveals the association between liver and skeletal muscle metabolites that have only recently been described in relation to exercise. In the mouse liver, continuous acute exercise was associated with significant increased abundance of putatively annotated (MSI level 2) ergothioneine (*p* < 0.01; FDR < 0.04) and decreased abundance of identified (MSI level 1) daidzein (*p* < 0.01; FDR < 0.04). Ergothioneine is a diet-derived antioxidant [54] that increases aerobic performance in rodent models [55] and accumulates in skeletal muscle mitochondria enhancing mitochondrial function in response to chronic endurance training [56]. Recent work has also shown that ergothioneine increases hydrogen sulfide production, leading to protein persulfidation and activation of cytosolic glycerol-3-phosphate dehydrogenase and subsequent NAD+ production [57]. While glycerol-3-phosphate levels were not altered in liver samples in the current study, there was a genotype/condition effect detected in skeletal muscle. Future work is warranted to confidently identify and quantify ergothioneine, and explore interactions between glycerol-3-phosphate and exercise. Daidzein is a phytoestrogen isoflavone commonly found in soybeans and that has been studied over the past years for its various suggested health-promoting properties [58]. However, the direct effects of continuous acute exercise on liver concentrations of daidzein have been uncharacterized prior to this present study. The biological meaning of decreased liver daidzein remains unknown. In gastrocnemius muscle, putatively annotated citicoline (MSI level 2) was significantly increased following continuous acute exercise (*p* < 0.01; FDR < 0.01). Citicoline, also known as cytidine-5′-diphosphocholine and CDP-choline, is an intermediate in the biosynthesis of phosphatidylcholine, the primary phospholipid in cell membranes. Citicoline has been associated with various neuroprotective effects, particularly when administered as a drug or dietary supplement, as reviewed elsewhere [59]. The mechanism responsible for increased skeletal muscle levels of citicoline in response to exercise and whether increased muscle citicoline is linked to additional health benefits remain to be elucidated. Since both citicoline and ergothioneine were only putatively annotated (MSI level 2) in the present study, targeted validation is required before drawing conclusions regarding their roles in acute exercise. Some of the top metabolites contributing to the separation of liver and skeletal muscle overall metabolite profiles are summarized in Table 1.

Some limitations associated with this study need to be acknowledged. Despite numerous advantages, untargeted metabolomics still faces some hurdles that have been described in the previous plasma metabolomic analysis [11] and reviewed elsewhere [10]. Briefly, the metabolites reported in the present study were restricted to the metabolite references available through in-house metabolite library and external databases, therefore limiting the scope of some biological interpretations. This study used a sample size based on power to detect differences in maximal running speed between genotypes and may therefore be insufficient to detect subtle effects in some specific metabolic pathways. To ensure sufficient blood volume collection for plasma metabolomic analyses in our previous study, CO_2_ exposure prior to euthanasia was utilized and may have potentially impacted plasma pH and metabolite concentrations, in both plasma and tissues. However, all mice were subjected to the same duration of CO_2_ exposure to minimize the likelihood of between-group differences. Furthermore, tissue samples were collected from mice in the fed state. If the timing of each mouse’s last food intake was different, this would potentially contribute to increased variance in metabolite levels observed between mice. Lastly, the exclusive use of male mice in this study did not permit detection of known and potential novel sex-related differences in metabolic responses to exercise in female mice, as recently reviewed [60]. Regardless of these limitations, the liver and skeletal muscle metabolomic datasets in the present study and complementary analyses of plasma metabolites detected known and novel metabolites associated with continuous acute exercise and mouse genotypes, adding to the limited body of evidence in the field of exercise metabolomics in mouse models [61,62] and contributing a large hypothesis-generating resource for future investigations by the exercise and metabolism fields.

## 5. Conclusions

In the present study, a total of 92 and 150 metabolites were identified/annotated in gastrocnemius muscle and liver, respectively, using untargeted MS-based metabolomics. Like the plasma metabolite responses across genotypes and conditions, metabolomic analyses indicated significant overall metabolite profile shifts between WT and DKI mice at rest and significant differences between rested and exercised conditions. In contrast to liver, an interaction effect was observed in skeletal muscle, suggesting differential muscle metabolite responses to continuous acute exercise between genotypes that more closely reflect the associated responses to exercise in plasma. Complementary bioenergetic analyses in permeabilized fibers further characterized the mouse DKI phenotype but suggest that differential metabolite responses to exercise between genotypes are likely unrelated to red and white gastrocnemius muscle mitochondrial bioenergetics. Collectively, these tissue metabolomic analyses have helped expand the knowledge base of metabolites associated with exercise and the disruption of AMPK-glycogen binding in vivo, revealing potential novel biomarkers that may contribute to exercise’s metabolic health benefits and the physiological effects of disrupting AMPK-glycogen interactions.

## Figures and Tables

**Table 1:** Key contributors to multivariate differences in overall tissue metabolite profiles.

Tissue	Canonical Variate (CV)	Metabolite Name	MSI ID	Cluster	Relative Abundance
Liver	CV1 (Condition Effect)	Cortisol	1	E	Exercise > Rest
		Adenosine monophosphate (AMP)	1	E	Exercise > Rest
		Ergothioneine	2	G	Exercise > Rest
		Acetylcholine	2	D	Rest > Exercise
		Daidzein	1	D	Rest > Exercise
	CV2 (Genotype Effect)	Histamine	1	B	DKI > WT
		Carbohydrate species ^a^	1	B	DKI > WT
		Methylimidazoleacetic acid	1	E	WT > DKI
		Creatine phosphate (PCr)	2	E	WT > DKI
		Adenosine monophosphate (AMP)	1	E	WT > DKI
Gastrocnemius Muscle	CV1 (Condition Effect)	Carnitine species ^b^	1	H	Exercise > Rest
		Adenosine	1	H	Exercise > Rest
		Inosine	1	H	Exercise > Rest
		Hypoxanthine	1	H	Exercise > Rest
		Xanthine	1	H	Exercise > Rest
		Inosine-5′-monophosophate (IMP)	2	H	Exercise > Rest
	CV2 (Genotype Effect)	N6,N6,N6-trimethyl-L-lysine	2	C	DKI > WT
		Creatine	1	B	DKI > WT
		Glutathione (reduced)	1	B	DKI > WT
		Glycerol-3-phosphate	1	G	Interaction GxC: WT > DKI at rest ^c^

^a^ Carhohydrate species: maltotriose, biose (disaccharide), stachyose; ^b^ Carnitine species: decanoylcarnitine, palmitoylcarnitine, propionylcarnitine, and putatively annotated hexanoylcarnitine (MSI level 2); ^c^ Cluster G metabolite demonstrated a significant genotype × condition interaction, with opposite changes in mean relative abundances following acute exercise (decreased in WT and increased in DKI gastrocnemius muscle).

## Data Availability

The original contributions presented in the study are included in the article/Supplementary Material, further inquiries can be directed to the corresponding authors.

## Supplementary Materials

The following supporting information can be downloaded at: https://www.mdpi.com/article/10.3390/metabo16030205/s1, Figure S1: Z-scores plot of the mean responses for each mouse liver metabolite cluster; Figure S2: Loading plot showing mouse liver metabolites that significantly contributed to CV1 and CV2; Figure S3: Z-scores plot of the mean responses for each mouse gastrocnemius muscle metabolite cluster; Figure S4: Loading plot showing mouse gastrocnemius muscle metabolites that significantly contributed to CV1 and CV2; Table S1: Summary of the metabolites identified/annotated from WT and DKI mouse liver; Table S2: Summary of the metabolites identified/annotated from WT and DKI mouse gastrocnemius muscle.
