# Supplementary material for: Remodeling of the Mouse Liver and Skeletal Muscle Metabolome in Response to Continuous Acute Exercise and Disruption of AMPK-Glycogen Interactions
Source: Metabolites. 2026 Mar 20;16(3):205. doi: 10.3390/metabo16030205 (PMC13028007; doi:10.3390/metabo16030205)
Supplement: Supplementary file 1 [file metabolites-16-00205-s001.zip › metabolites-4156446-supplementary.pdf]

Supplementary Material to:

Remodeling of the Mouse Liver and Skeletal Muscle Metabolome in Response to Continuous Acute Exercise and Disruption of AMPK-Glycogen Interactions

Mehdi R. Belhaj, David I. Broadhurst, Thomas Dignan, Jamie Whitfield, Lisa Murray-Segal, Naomi X. Y. Ling, Jonathan S. Oakhill, Bruce E. Kemp, John A. Hawley, Stacey N. Reinke and Nolan J. Hoffman

Table S1 – Summary of the metabolites identified/annotated from WT and DKI mouse liver

Column & Mode	Metabolite	Molecular Formula	Molecular Weight	RT (min)	RSD_{QC}	*P* value G	FDR G	*P* value C	FDR C	*P* value GxC	FDR GxC	MSI ID	Cluster
C18POS	Lithocholyltaurine	C26 H45 N O5 S	483.3016	4.65	2.634	0.618	0.600	0.914	0.635	0.908	1	1	A
C18POS	Bile Acid (C26 H45 N O6 S)	C26 H45 N O6 S	499.2968	4.11	2.875	0.182	0.373	0.636	0.565	0.686	1	1	A
C18POS	Bile Acid (C26 H45 N O7 S)	C26 H45 N O7 S	515.2923	3.85	2.409	0.160	0.348	0.440	0.461	0.699	1	1	A
C18NEG	Taurocholic acid	C26 H45 N O7 S	515.2917	3.87	1.448	0.146	0.338	0.467	0.475	0.817	1	1	A
C18NEG	Glycocholic acid	C26 H43 N O6	465.3092	4.30	0.471	0.417	0.506	0.379	0.439	0.264	1	1	A
C18NEG	Glycodeoxycholic acid	C26 H43 N O5	449.3143	4.97	5.454	0.524	0.571	0.765	0.608	0.171	1	2	A
C18NEG	Bile Acid (C24 H40 O4)	C24 H40 O4	392.2928	5.77	4.451	**0.010**	0.182	0.704	0.593	0.918	1	1	A
C18NEG	Cholic acid	C24 H40 O5	408.2879	4.79	2.458	0.119	0.354	0.855	0.624	0.654	1	2	A
C18NEG	β-Muricholic acid	C24 H40 O5	408.2881	4.52	2.002	0.296	0.439	0.673	0.577	0.642	1	2	A
C18NEG	Bile acid (C24 H40 O5)	C24 H40 O5	408.2876	4.06	7.872	0.578	0.588	0.299	0.392	0.828	1	2	A
C18NEG	Deoxycorticosterone 21-glucoside	C27 H40 O8	492.2699	4.23	11.973	0.994	0.708	0.609	0.566	0.349	1	2	A
C18POS	O-Phosphoserine	C3 H8 N O6 P	185.0091	0.84	2.618	0.924	0.731	**0.037**	0.109	0.360	1	1	A
C18NEG	Oxidized glutathione	C20 H32 N6 O12 S2	612.1518	0.92	2.062	0.736	0.660	0.557	0.533	0.905	1	1	B
C18POS	Spermidine	C7 H19 N3	145.1580	0.75	7.827	0.383	0.493	0.803	0.619	0.624	1	1	B
HILICPOS	Nicotinamide adenine dinucleotide (NAD+)	C21 H27 N7 O14 P2	663.1097	7.77	2.423	0.253	0.422	0.874	0.633	0.347	1	2	B
HILICPOS	Trans-urocanate	C6 H6 N2 O2	138.0428	1.52	8.167	0.617	0.605	0.060	0.144	0.713	1	1	B
HILICPOS	Arginine	C6 H14 N4 O2	174.1116	7.21	4.976	0.353	0.472	0.845	0.622	0.860	1	1	B
HILICPOS	N6,N6,N6-Trimethyl-L-lysine	C9 H20 N2 O2	188.1523	6.99	2.321	0.185	0.373	0.327	0.419	0.864	1	2	B
HILICPOS	Isoputreanine	C7 H16 N2 O2	160.1211	7.05	0.596	0.906	0.739	0.812	0.611	0.581	1	1	B

Continued

Table S1 – Continued

Column & Mode	Metabolite	Molecular Formula	Molecular Weight	RT (min)	RSD_{QC}	*P* value G	FDR G	*P* value C	FDR C	*P* value GxC	FDR GxC	MSI ID	Cluster
C18POS	Creatine	C4 H9 N3 O2	131.0695	0.89	1.344	0.193	0.375	0.959	0.643	0.902	1	1	B
C18POS	Histamine	C5 H9 N3	111.0798	0.78	2.436	**0.016**	0.186	0.623	0.563	0.914	1	1	B
C18NEG	Biose	C12 H22 O11	342.116	0.89	7.754	**0.017**	0.181	0.170	0.262	0.862	1	1	B
C18NEG	Maltotriose	C18 H32 O16	504.1684	0.9	14.402	**0.005**	0.177	0.822	0.614	0.103	1	1	B
C18NEG	Stachyose	C24 H42 O21	666.2216	0.91	16.097	0.059	0.244	0.431	0.456	**0.021**	1	1	B
HILICPOS	Decanoylcarnitine	C17 H33 N O4	315.2407	1.27	1.369	0.542	0.568	0.134	0.228	0.708	1	1	C
HILICPOS	5'-Methylthioadenosine	C11 H15 N5 O3 S	297.0893	1.22	0.472	0.934	0.728	**0.031**	0.103	0.737	1	1	C
HILICPOS	Pipecolic acid	C6 H11 N O2	129.0789	5.4	0.934	0.478	0.549	0.082	0.175	0.876	1	1	C
C18POS	Tyrosine	C9 H11 N O3	181.0739	1.01	2.002	0.457	0.531	**0.024**	**0.090**	0.057	1	1	C
HILICPOS	Dipalmitoylphosphatidylcholine	C40 H80 N O8 P	733.562	1.14	3.674	0.332	0.479	0.062	0.146	0.741	1	2	C
HILICPOS	Guanidoacetic acid	C3 H7 N3 O2	117.0538	5.93	3.263	0.535	0.577	0.490	0.488	0.640	1	2	C
HILICPOS	Hexanoylcarnitine	C13 H25 N O4	259.1782	1.76	1.132	0.066	0.235	0.927	0.635	0.355	1	2	C
C18POS	Glucosamine	C6 H13 N O5	179.0794	0.86	4.402	0.344	0.472	0.476	0.479	0.718	1	1	C
HILICPOS	Homoarginine hydrochloride	C7 H16 N4 O2	188.1272	7.09	4.666	0.945	0.721	0.389	0.441	0.384	1	2	C
C18NEG	Aspartic acid	C4 H7 N O4	133.0374	0.87	1.820	**0.018**	0.178	**0.024**	**0.088**	0.484	1	1	C
C18NEG	Pyruvic acid	C3 H4 O3	88.01596	0.92	2.884	**0.014**	0.188	0.075	0.169	0.734	1	1	C
C18NEG	Malic acid	C4 H6 O5	134.0214	0.92	2.884	**0.014**	0.215	0.075	0.173	0.734	1	1	C
C18NEG	Cis-Aconitic acid	C6 H6 O6	174.0164	0.99	4.970	**0.023**	0.189	0.117	0.205	0.913	1	1	C
HILICPOS	Anserine	C10 H16 N4 O3	240.1222	7.31	0.705	**0.044**	0.262	0.613	0.564	0.198	1	2	C
HILICPOS	Thiamine	C12 H16 N4 O S	264.1045	4.93	2.678	0.148	0.336	0.708	0.592	0.968	1	2	C

Continued

Table S1 – Continued

Column & Mode	Metabolite	Molecular Formula	Molecular Weight	RT (min)	RSD_{QC}	*P* value G	FDR G	*P* value C	FDR C	*P* value GxC	FDR GxC	MSI ID	Cluster
C18POS	2-Aminooctanoic acid	C8 H17 N O2	159.1259	3.47	1.112	**0.047**	0.230	0.358	0.436	0.816	1	1	C
C18POS	Pyroglutamic acid	C5 H7 N O3	129.0427	0.85	2.370	0.549	0.569	0.513	0.506	0.669	1	1	C
C18POS	Citrulline	C6 H13 N3 O3	175.0957	0.86	6.255	0.062	0.238	0.084	0.176	0.311	1	1	D
HILICPOS	Cystathionine	C7 H14 N2 O4 S	222.0674	7.82	4.636	0.980	0.717	0.098	0.186	0.982	1	1	D
C18POS	Alanine	C3 H7 N O2	89.04776	0.84	2.050	0.992	0.716	**0.001**	**0.018**	0.803	1	1	D
HILICPOS	N-Acetyl-L-arginine	C8 H16 N4 O3	216.1221	5.88	4.036	0.742	0.660	**0.006**	**0.037**	0.376	1	2	D
HILICPOS	Imidazoleacetic acid	C5 H6 N2 O2	126.0428	5.68	11.934	0.932	0.732	**0.016**	**0.066**	0.476	1	1	D
C18NEG	Daidzein	C15 H10 O4	254.0579	3.89	3.445	0.803	0.686	**0.007**	**0.033**	0.986	1	1	D
C18NEG	Phenylpropionylglycine	C11 H13 N O3	207.0894	3.70	9.023	0.344	0.477	0.243	0.363	0.646	1	1	D
C18NEG	5-Methoxyindoleacetate	C11 H11 N O3	205.0737	3.75	3.974	0.307	0.450	0.665	0.580	0.902	1	1	D
C18POS	Phenylacetylglycine	C10 H11 N O3	193.074	3.48	6.440	0.975	0.723	0.980	0.648	0.680	1	1	D
C18NEG	Indoxyl sulfate	C8 H7 N O4 S	213.0096	3.23	4.122	0.285	0.442	0.934	0.635	0.786	1	1	D
HILICPOS	Propionylcarnitine	C10 H19 N O4	217.1312	3.27	3.405	0.201	0.377	0.051	0.135	0.519	1	1	D
HILICPOS	Acetylcholine	C7 H15 N O2	145.1101	4.40	5.142	0.834	0.701	**1.96E-04**	**0.004**	0.832	1	2	D
C18POS	Methionine	C5 H11 N O2 S	149.051	0.94	5.329	0.137	0.349	**0.033**	0.106	0.501	1	1	D
C18POS	Serine	C3 H7 N O3	105.0426	0.84	4.009	0.367	0.483	0.057	0.144	0.130	1	1	D
HILICPOS	Threonine/Homoserine	C4 H9 N O3	119.0582	6.15	1.521	0.735	0.666	0.164	0.256	0.274	1	1	D
HILICPOS	Cytidine 5′-monophosphate	C9 H14 N3 O8 P	323.0518	7.99	1.546	0.536	0.572	0.294	0.397	0.425	1	2	E
HILICPOS	Citicoline	C14 H26 N4 O11 P2	488.1075	7.90	1.358	0.161	0.345	0.876	0.630	0.567	1	2	E
HILICPOS	Adenosine monophosphate	C10 H14 N5 O7 P	347.0632	6.72	0.864	0.199	0.379	**0.010**	**0.047**	0.954	1	1	E

Continued

Table S1 – Continued

Column & Mode	Metabolite	Molecular Formula	Molecular Weight	RT (min)	RSD_{QC}	*P* value G	FDR G	*P* value C	FDR C	*P* value GxC	FDR GxC	MSI ID	Cluster
C18NEG	Glucosamine 6-phosphate	C6 H14 N O8 P	259.0456	0.80	1.992	0.053	0.245	0.053	0.137	0.789	1	1	E
HILICPOS	Creatine phosphate	C4 H10 N3 O5 P	211.0357	7.22	0.334	**0.046**	0.236	0.095	0.188	0.936	1	2	E
HILICPOS	Guanidinoethyl sulfonate	C3 H9 N3 O3 S	167.0363	5.40	1.508	**0.020**	0.180	0.304	0.395	0.848	1	2	E
HILICPOS	Methylimidazoleacetic acid	C6 H8 N2 O2	140.0583	4.89	7.477	**0.005**	0.139	**0.017**	**0.069**	0.195	1	1	E
HILICPOS	p-Aminobenzoic acid	C7 H7 N O2	137.0476	5.22	1.761	0.136	0.353	0.883	0.631	0.822	1	1	E
HILICPOS	Lysine	C6 H14 N2 O2	146.1054	7.35	2.007	0.819	0.694	0.371	0.441	0.688	1	1	E
C18POS	Niacinamide	C6 H6 N2 O	122.0481	0.91	2.482	0.167	0.349	0.331	0.419	0.520	1	1	E
HILICPOS	1-Methylnicotinamide	C7 H8 N2 O	136.0635	3.77	1.063	0.141	0.343	**0.002**	**0.019**	0.259	1	2	E
C18NEG	Cyclic ADP-ribose	C15 H21 N5 O13 P2	541.0605	0.92	3.719	0.907	0.734	**0.026**	**0.087**	0.091	1	2	E
C18NEG	Uridine 5'-diphosphoglucuronic acid	C15 H22 N2 O18 P2	580.0339	0.96	6.895	0.707	0.656	**0.040**	0.112	0.940	1	2	E
HILICPOS	1-Methyladenosine	C11 H15 N5 O4	281.1123	5.13	2.007	0.975	0.718	**0.001**	**0.018**	0.393	1	2	E
C18NEG	Steroid hormone (C21 H30 O4)	C21 H30 O4	346.2144	4.62	7.216	0.282	0.450	**1.98E-16**	**1.95E-14**	0.621	1	2	E
C18POS	Cortisol	C21 H30 O5	362.2096	4.10	2.358	**0.029**	0.205	**1.08E-08**	**5.31E-07**	**0.019**	1	1	E
C18NEG	Uridine diphosphate-hexose	C15 H24 N2 O17 P2	566.0553	0.80	3.440	**0.003**	0.141	**1.52E-05**	**0.001**	**0.047**	1	1	E
C18NEG	Myristyl sulfate	C14 H30 O4 S	294.1867	5.94	7.473	0.350	0.474	0.084	0.173	0.265	1	2	E
C18NEG	Hexose	C6 H12 O6	180.0633	0.87	2.244	0.225	0.400	**0.006**	**0.038**	0.131	1	1	E
HILICPOS	Carnosine	C9 H14 N4 O3	226.1065	7.37	0.548	**0.029**	0.219	0.126	0.218	0.619	1	1	E
HILICPOS	Glycerol 3-phosphate	C3 H9 O6 P	172.0135	6.65	0.534	0.057	0.246	0.657	0.578	0.836	1	1	F
C18POS	Uracil	C4 H4 N2 O2	112.0273	1.01	12.031	0.895	0.741	0.805	0.615	0.349	1	1	F
C18NEG	Uric acid	C5 H4 N4 O3	168.0282	1.27	1.013	0.454	0.532	0.830	0.615	0.884	1	1	F

Continued

Table S1 – Continued

Column & Mode	Metabolite	Molecular Formula	Molecular Weight	RT (min)	RSD_{QC}	*P* value G	FDR G	*P* value C	FDR C	*P* value GxC	FDR GxC	MSI ID	Cluster
C18NEG	Xanthosine	C10 H12 N4 O6	284.0756	2.90	4.018	0.247	0.419	0.554	0.535	0.870	1	2	F
C18POS	Xanthine	C5 H4 N4 O2	152.0333	1.01	1.773	0.336	0.478	0.713	0.591	0.968	1	1	F
HILICPOS	Adenosine	C10 H13 N5 O4	267.0965	2.27	2.644	0.512	0.582	0.885	0.627	0.755	1	1	F
C18NEG	Inosine	C10 H12 N4 O5	268.0807	0.94	2.676	0.290	0.442	0.551	0.537	0.932	1	1	F
C18NEG	Ribulose 5-phosphate	C5 H11 O8 P	230.0191	0.81	4.002	0.903	0.742	0.422	0.457	0.289	1	1	F
C18NEG	Inosine-5'-monophosphate (IMP)	C10 H13 N4 O8 P	348.047	0.92	0.543	0.400	0.497	0.747	0.604	0.229	1	2	F
HILICPOS	Nicotinamide	C6 H6 N2 O	122.0479	5.15	1.924	0.514	0.572	0.161	0.255	0.156	1	1	F
C18NEG	Hexose-phosphate	C6 H13 O9 P	260.0297	0.80	0.862	0.936	0.724	**0.001**	**0.011**	0.413	1	1	F
C18POS	Glucose 6-phosphate	C6 H13 O9 P	260.0301	0.79	1.020	0.709	0.653	**0.002**	**0.018**	0.755	1	1	F
C18POS	Adenine	C5 H5 N5	135.0545	0.90	3.035	0.513	0.577	**0.003**	**0.026**	0.355	1	1	F
HILICPOS	Hypoxanthine	C5 H4 N4 O	136.0384	2.19	2.498	0.553	0.568	**0.006**	**0.040**	0.465	1	1	F
HILICPOS	Guanosine monophosphate	C10 H14 N5 O8 P	363.058	7.35	4.010	0.754	0.665	**0.046**	0.125	0.891	1	1	F
C18NEG	Uridine monophosphate (UMP)	C9 H13 N2 O9 P	324.0355	0.92	3.746	0.523	0.576	0.296	0.394	0.902	1	2	F
C18NEG	Asparagine	C4 H8 N2 O3	132.0534	0.86	2.031	0.668	0.642	0.699	0.594	0.994	1	1	F
C18POS	N-acetyltryptophan	C13 H14 N2 O3	246.1006	3.64	3.140	0.096	0.294	**0.015**	**0.062**	0.561	1	1	F
HILICPOS	Tryptophan	C11 H12 N2 O2	204.0897	4.60	2.687	0.783	0.680	0.217	0.330	0.957	1	1	F
C18NEG	Gulonolactone	C6 H10 O6	178.0476	0.88	4.068	0.090	0.299	0.259	0.381	0.586	1	1	F
C18NEG	Gluconic acid	C6 H12 O7	196.0581	0.87	2.498	**0.045**	0.251	0.059	0.145	0.772	1	2	F
HILICPOS	Ergothioneine	C9 H15 N3 O2 S	229.0884	5.70	1.917	0.214	0.387	**0.006**	**0.034**	0.396	1	2	G
C18NEG	Saccharopine	C11 H20 N2 O6	276.1319	0.88	0.678	0.208	0.383	0.406	0.449	0.233	1	1	G

Continued

Table S1 – Continued

Column & Mode	Metabolite	Molecular Formula	Molecular Weight	RT (min)	RSD_{QC}	*P* value G	FDR G	*P* value C	FDR C	*P* value GxC	FDR GxC	MSI ID	Cluster
C18NEG	Cinnamoylglycine	C11 H11 N O3	205.0737	3.67	4.200	0.694	0.656	**0.012**	**0.052**	0.996	1	1	G
HILICPOS	Hypotaurine	C2 H7 N O2 S	109.0198	5.90	0.203	0.283	0.445	0.366	0.440	0.396	1	1	G
HILICPOS	Melatonin	C13 H16 N2 O2	232.1209	0.98	9.075	0.094	0.303	0.453	0.465	0.761	1	1	G
C18POS	Glycine	C2 H5 N O2	75.03205	0.84	3.586	0.094	0.296	0.142	0.233	0.264	1	1	G
HILICPOS	Phenylethylamine	C8 H11 N	121.0889	0.84	4.323	0.965	0.726	0.354	0.436	0.898	1	1	G
HILICPOS	Glutathione	C10 H17 N3 O6 S	307.0837	6.89	1.331	0.991	0.720	0.291	0.399	0.696	1	1	G
C18NEG	Taurine	C2 H7 N O3 S	125.0146	0.87	1.148	0.395	0.502	0.559	0.529	0.326	1	2	G
C18NEG	S-Adenosylhomocysteine	C14 H20 N6 O5 S	384.1216	1.91	0.206	0.615	0.608	0.591	0.555	0.734	1	1	G
HILICPOS	Beta-Alanine	C3 H7 N O2	89.04767	5.71	5.481	0.948	0.718	0.264	0.377	0.870	1	1	G
C18POS	Carnitine	C7 H15 N O3	161.1052	0.88	0.525	0.133	0.363	0.080	0.175	0.254	1	1	G
HILICPOS	Leucine/Isoleucine	C6 H13 N O2	131.0945	4.48	4.093	0.134	0.358	0.971	0.647	0.873	1	1	G
HILICPOS	Acetylcarnitine	C9 H17 N O4	203.1154	4.21	2.277	0.913	0.727	0.159	0.256	0.532	1	1	G
C18NEG	Benzoic acid	C7 H6 O2	122.0368	3.53	7.324	0.968	0.723	0.440	0.457	0.781	1	2	H
HILICPOS	2-Aminoisobutyric acid	C4 H9 N O2	103.0633	5.63	2.267	0.069	0.239	0.624	0.559	0.365	1	1	H
C18POS	Cytosine	C4 H5 N3 O	111.0433	3.31	4.194	0.936	0.719	0.913	0.638	0.646	1	2	H
HILICPOS	Bilirubin	C33 H36 N4 O6	584.2627	0.63	8.849	0.699	0.654	0.673	0.582	0.235	1	2	H
C18POS	Cis-4,7,10,13,16,19-Docosahexaenoic acid	C22 H32 O2	328.2400	8.29	5.613	0.295	0.443	0.393	0.440	0.510	1	2	H
C18POS	Arachidonic acid	C20 H32 O2	304.2401	8.47	10.203	0.377	0.491	0.377	0.442	0.477	1	2	H
C18POS	1-Linoleoyl glycerol	C21 H38 O4	354.277	7.96	4.787	0.130	0.376	0.263	0.382	0.529	1	2	H
HILICPOS	4-Guanidinobutyric acid	C5 H11 N3 O2	145.0851	4.47	2.343	0.233	0.401	**0.006**	**0.033**	0.361	1	2	H

Continued

Table S1 – Continued

Column & Mode	Metabolite	Molecular Formula	Molecular Weight	RT (min)	RSD_{QC}	*P* value G	FDR G	*P* value C	FDR C	*P* value GxC	FDR GxC	MSI ID	Cluster
HILICPOS	N-Acetylneuraminic acid	C11 H19 N O9	309.1058	6.89	4.452	0.781	0.683	0.282	0.392	0.570	1	1	H
HILICPOS	3-Methylhistidine	C7 H11 N3 O2	169.085	7.17	4.628	0.803	0.691	0.343	0.428	0.917	1	1	H
HILICPOS	Y-Glutamylcysteine	C8 H14 N2 O5 S	250.0622	6.73	1.620	0.225	0.395	0.622	0.567	0.921	1	2	H
HILICPOS	Palmitoyl sphingomyelin	C39 H79 N2 O6 P	702.5676	0.78	2.434	0.673	0.642	0.096	0.186	0.881	1	2	H
HILICPOS	N3,N4-Dimethyl-L-arginine	C8 H18 N4 O2	202.1428	6.66	2.193	0.538	0.569	**0.006**	**0.036**	0.452	1	2	H
HILICPOS	Trimethylamine N-oxide	C3 H9 N O	75.06834	2.68	1.519	0.151	0.336	**0.025**	**0.087**	0.535	1	1	H
C18POS	Cytidine	C9 H13 N3 O5	243.0855	0.90	6.038	0.145	0.344	0.762	0.611	0.391	1	1	H
HILICPOS	Proline	C5 H9 N O2	115.0633	5.33	1.898	0.342	0.480	0.729	0.594	0.208	1	2	H
HILICPOS	N-Alpha-acetyllysine	C8 H16 N2 O3	188.116	6.00	0.521	0.438	0.526	0.990	0.650	0.420	1	1	H
HILICPOS	Uridine diphosphate-N-acetylglucosamine	C17 H27 N3 O17 P2	607.0814	8.05	2.481	0.604	0.603	0.910	0.641	0.699	1	1	H
C18NEG	9(Z),11(E)-Conjugated linoleic acid	C18 H32 O2	280.2401	8.89	2.109	0.994	0.713	**0.035**	0.107	0.653	1	2	H
HILICPOS	Palmitoylcarnitine	C23 H45 N O4	399.3342	1.08	0.674	0.716	0.654	**0.005**	**0.039**	0.741	1	1	H
HILICPOS	S-Adenosylmethionine	C15 H22 N6 O5 S	398.1375	7.62	3.534	0.843	0.704	**0.002**	**0.017**	0.336	1	2	H
C18NEG	Suberic acid	C8 H14 O4	174.0892	3.37	1.674	0.445	0.528	**6.18E-05**	**0.002**	0.668	1	1	H
C18NEG	Sphingosine 1-phosphate	C18 H38 N O5 P	379.2488	5.86	3.248	0.413	0.507	0.282	0.397	0.359	1	1	H
C18NEG	ADP	C10 H15 N5 O10 P2	427.0296	0.92	6.443	**0.033**	0.221	0.714	0.587	0.811	1	1	H
C18NEG	Flavin adenine dinucleotide (FAD)	C27 H33 N9 O15 P2	785.1587	3.18	1.504	**0.002**	0.227	**0.036**	0.109	0.616	1	2	H
C18NEG	Flavin mononucleotide (FMN)	C17 H21 N4 O9 P	456.1049	3.22	1.331	**0.045**	0.241	0.092	0.184	0.098	1	2	H
HILICPOS	Pantothenic acid	C9 H17 N O5	219.1105	1.45	2.975	0.064	0.237	0.421	0.461	0.186	1	1	I
HILICPOS	Aminoadipic acid	C6 H11 N O4	161.0687	6.16	3.915	**0.006**	0.126	0.106	0.193	0.169	1	2	I

Continued

Table S1 – Continued

Column & Mode	Metabolite	Molecular Formula	Molecular Weight	RT (min)	RSD_{QC}	*P* value G	FDR G	*P* value C	FDR C	*P* value GxC	FDR GxC	MSI ID	Cluster
HILICPOS	Glutamine	C5 H10 N2 O3	146.0690	6.36	2.721	0.057	0.254	0.768	0.605	0.755	1	1	I
HILICPOS	Betaine	C5 H11 N O2	117.0788	4.72	1.770	0.141	0.351	0.426	0.456	0.693	1	1	I
HILICPOS	Glutamic acid	C5 H9 N O4	147.0530	6.42	1.640	0.059	0.235	0.947	0.639	0.923	1	1	I
HILICPOS	Xanthurenic acid	C10 H7 N O4	205.0370	4.10	1.934	0.396	0.497	0.807	0.612	0.386	1	2	I
HILICPOS	Stearoylethanolamide	C20 H41 N O2	327.3133	0.65	5.965	**0.043**	0.273	0.782	0.611	0.494	1	1	I
HILICPOS	1-Methylhistidine	C7 H11 N3 O2	169.0850	6.75	0.842	0.579	0.583	0.136	0.226	0.730	1	1	I
HILICPOS	Oleamide	C18 H35 N O	281.2715	0.71	1.817	0.131	0.367	0.099	0.185	0.966	1	1	I
HILICPOS	Riboflavin	C17 H20 N4 O6	376.1379	3.56	3.312	0.187	0.369	0.114	0.205	0.982	1	2	I
HILICPOS	Phenylalanine	C9 H11 N O2	165.0788	4.57	1.834	0.254	0.418	0.920	0.634	0.370	1	1	I
HILICPOS	Jasmonic acid	C12 H18 O3	210.1254	0.67	7.106	0.275	0.446	0.795	0.617	0.282	1	1	I
HILICPOS	Histidine	C6 H9 N3 O2	155.0694	7.28	2.176	0.911	0.731	0.385	0.441	0.766	1	1	I

Table S1 – A total of 150 mouse liver metabolites were identified/annotated, including 29 metabolites significantly different between conditions (i.e., exercise versus rest) based on univariate analysis. Statistically significant values ($P < 0.05$ and/or FDR < 0.1) appear in bold text. C: condition; G: genotype; G x C: interaction between condition and genotype; FDR: false discovery rate; MSI: Metabolomics Standards Initiative; RSD_{QC}: relative standard deviation in quality control samples; RT: retention time. Metabolites in this table are sorted by cluster.

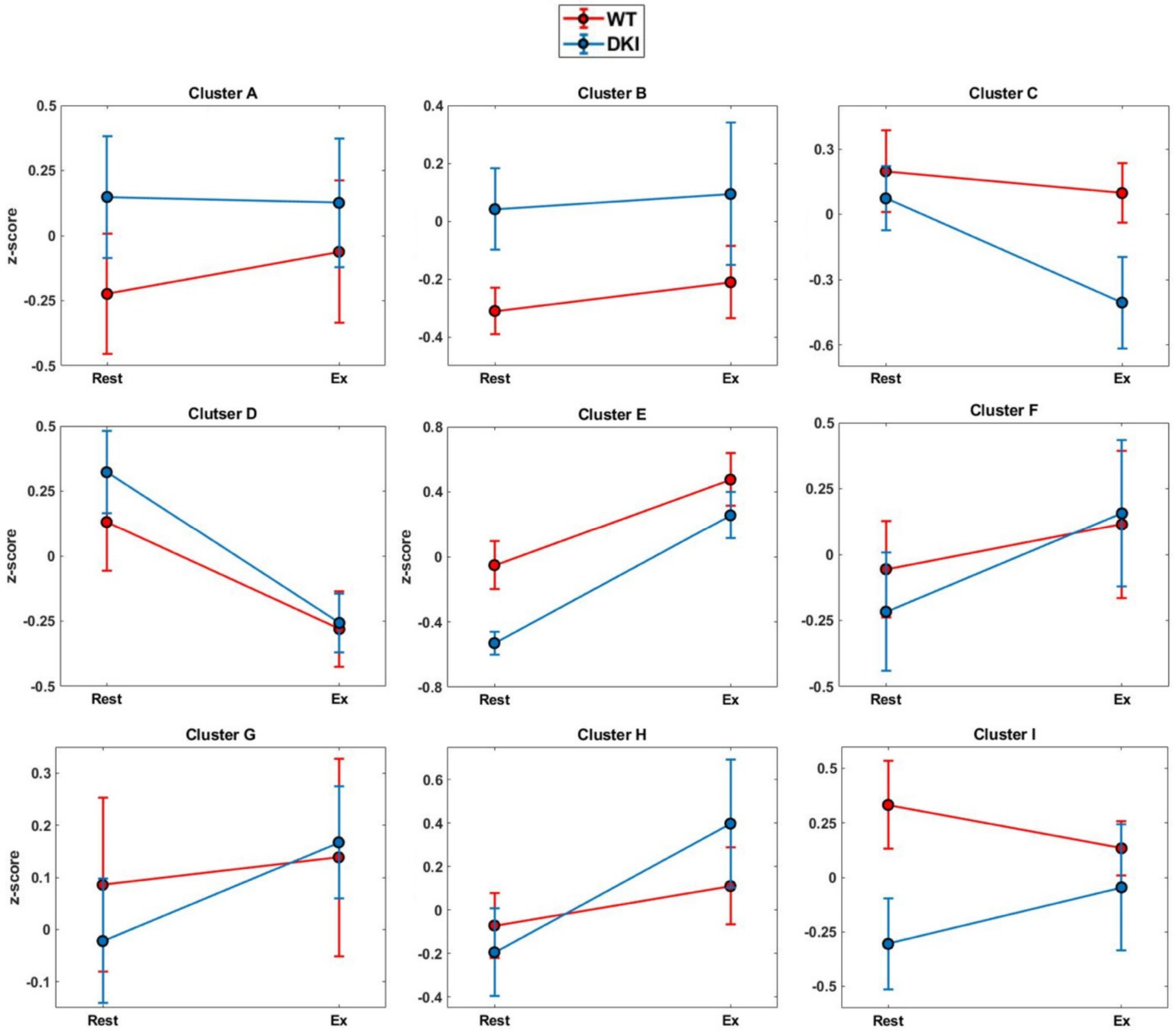

Figure S1 – Z-scores plot of the mean responses for each mouse liver metabolite cluster. After conversion of individual metabolite $\log_{10}$ responses to a z-score, the average response of each cluster was calculated and presented as a group mean with error bars indicating the standard error (Red = WT; Blue = DKI). Following two-way ANOVA, Clusters B and E showed significant differences in the average group metabolite abundance between genotypes. Cluster B showed a significantly increased abundance in DKI relative to WT mice ($P < 0.04$), while Cluster E showed a significantly decreased average group metabolite abundance ($P < 0.02$) in DKI compared to WT mice. Cluster E also showed significantly increased metabolite abundance associated with exercise ($P = 1.13 \times 10^{-5}$). In contrast, Cluster D showed a significantly decreased average group metabolite abundance in response to exercise ($P < 0.01$).

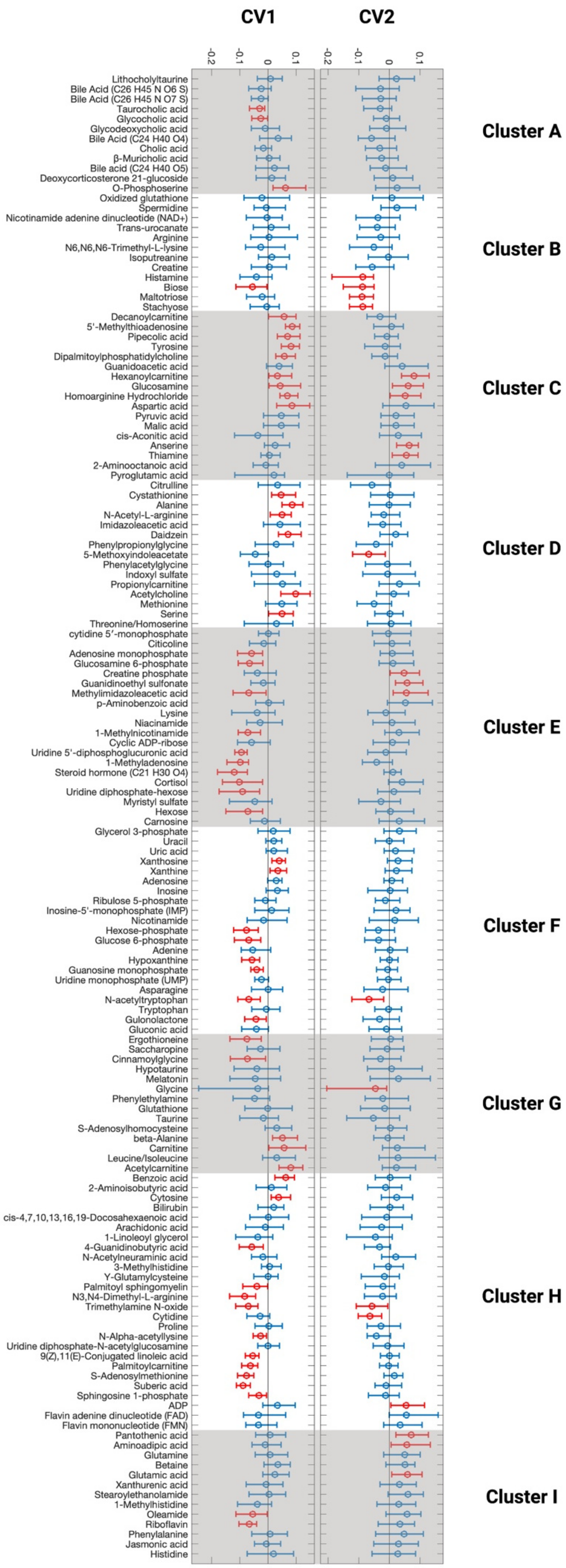

Figure S2 – Related to **Figure 2**. Loading plot showing mouse liver metabolites that significantly contributed to CV1 and CV2. Data are represented as mean loading values within 95% CI. Metabolites that significantly contributed to CV1 and/or CV2 are represented by red-colored dots and CIs, while blue dots and CIs represent metabolites that did not significantly contribute to their respective CV. Metabolites are sorted according to HCA clusters A to I (top-down). CI: confidence interval, CV: canonical variate, HCA: hierarchical cluster analysis.

Table S2 – Summary of the metabolites identified/annotated from WT and DKI mouse gastrocnemius muscle

Column & Mode	Metabolite	Molecular Formula	Molecular Weight	RT (min)	RSD_{QC}	*P* value G	FDR G	*P* value C	FDR C	*P* value GxC	FDR GxC	MSI ID	Cluster
C18NEG	Adenosine triphosphate (ATP)	C10 H16 N5 O13 P3	506.9954	1.17	6.429	0.902	0.833	0.550	0.527	0.144	0.523	2	A
C18POS	Spermidine	C7 H19 N3	145.1579	0.75	9.513	0.196	0.563	0.767	0.604	**0.020**	0.804	1	A
C18POS	Spermine	C10 H26 N4	202.2157	0.74	0.710	**0.027**	0.413	0.135	0.312	**0.023**	0.464	1	A
HILICPOS	Melatonin	C13 H16 N2 O2	232.1215	0.99	3.546	0.491	0.778	0.296	0.428	0.630	0.435	1	A
C18POS	Adenosine monophosphate	C10 H14 N5 O7 P	347.0630	1.00	2.875	0.554	0.843	0.101	0.263	0.912	0.440	1	A
HILICPOS	1-Methyladenosine	C11 H15 N5 O4	281.1125	5.09	2.922	0.883	0.835	**0.035**	0.115	0.930	0.429	2	A
HILICPOS	1-Methylhistidine	C7 H11 N3 O2	169.0853	6.70	4.518	0.732	0.847	0.178	0.370	0.914	0.436	1	A
HILICPOS	Cytosine	C4 H5 N3 O	111.0434	3.28	11.840	0.763	0.822	0.362	0.480	0.697	0.430	2	A
HILICPOS	2-Aminoisobutyric acid	C4 H9 N O2	103.0635	5.43	1.212	0.953	0.822	0.992	0.671	0.921	0.434	1	A
HILICPOS	p-Aminobenzoic acid	C7 H7 N O2	137.0479	4.86	4.435	0.931	0.850	0.306	0.433	0.148	0.495	1	A
HILICPOS	3-Hydroxyanthranilic acid	C7 H7 N O3	153.0427	1.28	3.919	0.933	0.842	0.269	0.429	0.927	0.432	1	A
HILICPOS	1-Methylnicotinamide	C7 H8 N2 O	136.0638	3.72	3.849	0.394	0.711	0.993	0.665	0.377	0.458	2	A
HILICPOS	Palmitoyl sphingomyelin	C39 H79 N2 O6 P	702.5692	1.60	5.303	0.779	0.785	0.472	0.533	0.674	0.451	2	A
HILICPOS	Pantothenic acid	C9 H17 N O5	219.1109	1.45	4.396	0.775	0.802	0.415	0.496	0.624	0.439	1	A
HILICPOS	5'-Methylthioadenosine	C11 H15 N5 O3 S	297.0901	1.20	2.768	0.776	0.793	0.117	0.280	0.825	0.447	1	A
HILICPOS	Dipalmitoylphosphatidylcholine	C40 H80 N O8 P	733.5635	0.76	2.851	0.941	0.830	0.630	0.568	0.939	0.418	2	A
C18POS	Histidine	C6 H9 N3 O2	155.0695	0.83	0.156	0.204	0.529	0.066	0.206	0.207	0.518	1	B
HILICPOS	3-Methylhistidine	C7 H11 N3 O2	169.0853	7.28	1.881	**0.018**	0.346	0.835	0.634	0.587	0.444	1	B
C18POS	Taurine	C2 H7 N O3 S	125.0147	0.86	0.784	**0.044**	0.431	0.976	0.667	0.180	0.515	2	B

Continued

Table S2 – Continued

Column & Mode	Metabolite	Molecular Formula	Molecular Weight	RT (min)	RSD_{QC}	*P* value G	FDR G	*P* value C	FDR C	*P* value GxC	FDR GxC	MSI ID	Cluster
C18POS	Carnosine	C9 H14 N4 O3	226.1066	0.85	0.294	0.060	0.425	0.765	0.610	0.786	0.457	1	B
HILICPOS	Creatine	C4 H9 N3 O2	131.0696	6.12	2.770	0.088	0.427	0.696	0.602	0.368	0.476	1	B
C18POS	Proline	C5 H9 N O2	115.0634	0.9	1.685	0.093	0.361	0.479	0.533	0.959	0.413	2	B
C18POS	Hydroxyproline	C5 H9 N O3	131.0583	0.86	6.750	**0.029**	0.381	0.340	0.460	0.667	0.453	2	B
C18POS	Glutathione	C10 H17 N3 O6 S	307.084	0.91	1.942	0.074	0.439	0.277	0.420	0.803	0.460	1	B
C18POS	D2-Aminooctanoic acid	C8 H17 N O2	159.1259	3.47	0.661	0.690	0.878	0.835	0.626	0.904	0.453	1	B
C18NEG	2-Hydroxycaproic acid	C6 H12 O3	132.0786	3.50	3.270	0.284	0.629	0.086	0.244	0.813	0.459	1	B
HILICPOS	Isoleucine/Leucine	C6 H13 N O2	131.0949	4.57	2.654	0.687	0.904	**3.05E-04**	**0.003**	0.133	0.669	1	B
C18POS	Carnitine	C7 H15 N O3	161.1051	0.88	1.226	0.050	0.392	**4.21E-07**	**2.65E-05**	0.712	0.426	1	C
HILICPOS	Ornithine	C5 H12 N2 O2	132.0900	7.37	1.033	0.971	0.828	**0.006**	**0.030**	0.458	0.417	1	C
HILICPOS	Gamma-Aminobutyric acid	C4 H9 N O2	103.0635	5.22	4.526	0.575	0.843	0.221	0.381	0.077	0.516	1	C
C18POS	Niacinamide	C6 H6 N2 O	122.0480	1.02	1.458	0.356	0.674	0.743	0.625	0.958	0.417	1	C
HILICPOS	Guanidinoethyl sulfonate	C3 H9 N3 O3 S	167.0365	5.36	5.833	0.083	0.461	0.934	0.653	0.435	0.459	2	C
HILICPOS	Trimethylamine N-oxide	C3 H9 N O	75.0685	2.69	4.638	0.572	0.854	0.430	0.505	0.324	0.542	1	C
C18POS	Histamine	C5 H9 N3	111.0797	0.79	0.044	0.720	0.859	0.215	0.393	0.584	0.459	1	C
HILICPOS	Isoputreanine	C7 H16 N2 O2	160.1214	7.02	2.952	0.736	0.840	0.636	0.565	0.688	0.431	1	C
HILICPOS	Arginine	C6 H14 N4 O2	174.1119	7.18	0.680	0.337	0.671	0.594	0.552	0.286	0.520	1	C
C18POS	Lysine	C6 H14 N2 O2	146.1055	0.80	11.756	0.139	0.492	0.541	0.526	0.441	0.442	1	C
C18POS	Pipecolic acid	C6 H11 N O2	129.079	0.80	5.815	0.147	0.495	0.611	0.559	0.599	0.429	1	C
C18POS	Threonine	C4 H9 N O3	119.0582	0.92	5.188	0.219	0.548	0.104	0.259	0.294	0.513	1	C

Continued

Table S2 – Continued

Column & Mode	Metabolite	Molecular Formula	Molecular Weight	RT (min)	RSD_{QC}	*P* value G	FDR G	*P* value C	FDR C	*P* value GxC	FDR GxC	MSI ID	Cluster
HILICPOS	N6,N6,N6-Trimethyl-L-lysine	C9 H20 N2 O2	188.1527	6.95	3.794	**4.82E-04**	**0.037**	0.750	0.623	0.372	0.466	2	C
HILICPOS	Trans-urocanate	C6 H6 N2 O2	138.0431	1.51	6.674	**0.016**	0.412	0.089	0.239	0.568	0.456	1	C
HILICPOS	Guanine	C5 H5 N5 O	151.0496	3.99	2.364	**0.038**	0.426	0.193	0.388	0.455	0.424	2	C
HILICPOS	Thiamine	C12 H16 N4 O S	264.1048	4.89	3.803	0.677	0.906	0.138	0.306	0.686	0.444	2	D
C18POS	5-Methoxyindoleacetate	C11 H11 N O3	205.0739	3.72	9.455	0.153	0.476	0.842	0.624	0.439	0.451	1	D
C18NEG	Cinnamoylglycine	C11 H11 N O3	205.0737	3.73	5.434	0.089	0.405	0.762	0.615	0.467	0.416	1	D
C18NEG	Phenylacetylglycine	C10 H11 N O3	193.0738	3.48	2.489	0.197	0.545	0.863	0.632	0.896	0.455	1	D
C18NEG	Indoxyl sulfate	C8 H7 N O4 S	213.0095	3.21	1.360	0.109	0.402	0.195	0.378	0.872	0.448	1	D
HILICPOS	Methionine	C5 H11 N O2 S	149.0512	4.94	3.812	0.940	0.839	0.239	0.402	0.550	0.450	1	E
HILICPOS	Adenine	C5 H5 N5	135.0548	7.72	4.675	**0.046**	0.395	0.540	0.534	0.225	0.502	1	E
C18POS	Glucose 6-phosphate	C6 H13 O9 P	260.0300	0.79	6.172	0.755	0.837	0.271	0.422	0.059	0.593	2	E
C18POS	Creatine phosphate	C4 H10 N3 O5 P	211.0358	1.05	7.978	0.338	0.657	0.409	0.499	0.494	0.431	2	E
HILICPOS	Glutathione oxidized	C20 H32 N6 O12 S2	612.1529	8.47	0.528	0.637	0.883	0.665	0.582	0.213	0.501	2	E
C18POS	Glycine	C2 H5 N O2	75.03197	0.84	9.872	0.772	0.810	0.151	0.323	0.905	0.448	1	E
C18POS	Bile Acid (C26 H45 N O7 S)	C26 H45 N O7 S	515.2919	3.85	0.073	0.226	0.532	0.405	0.504	0.934	0.421	1	E
C18NEG	Bile acid (C26 H45 N O6 S)	C26 H45 N O6 S	499.2973	4.18	3.741	0.398	0.702	0.384	0.487	0.817	0.449	1	E
C18NEG	Glycocholic acid	C26 H43 N O6	465.3094	4.28	4.091	0.316	0.682	0.291	0.431	0.586	0.452	1	E
HILICPOS	2-Hydroxycinnamic acid	C9 H8 O3	164.0472	5.32	4.010	0.198	0.529	0.761	0.623	0.906	0.443	1	F
HILICPOS	Tyrosine	C9 H11 N O3	181.0740	5.32	0.456	0.243	0.554	0.445	0.513	0.931	0.424	1	F
C18POS	Phenylalanine	C9 H11 N O2	165.0791	2.94	3.264	0.876	0.840	0.367	0.476	0.836	0.435	1	F

Continued

Table S2 – Continued

Column & Mode	Metabolite	Molecular Formula	Molecular Weight	RT (min)	RSD_{QC}	*P* value G	FDR G	*P* value C	FDR C	*P* value GxC	FDR GxC	MSI ID	Cluster
HILICPOS	Asparagine	C4 H8 N2 O3	132.0536	6.44	2.103	0.671	0.914	0.522	0.532	0.830	0.443	1	F
HILICPOS	Hypotaurine	C2 H7 N O2 S	109.0200	5.86	1.834	1.000	0.844	0.815	0.626	0.525	0.438	1	F
C18POS	Sugar alcohol (C6H14O6)	C6 H14 O6	182.0789	0.86	1.040	0.634	0.894	0.815	0.634	0.351	0.502	1	F
C18NEG	Bile acid (C24 H40 O5)	C24 H40 O5	408.2877	4.76	1.471	0.455	0.751	0.903	0.646	0.229	0.483	2	F
C18NEG	Deoxycholic acid	C24 H40 O4	392.2926	5.74	5.046	0.411	0.710	0.496	0.541	0.197	0.527	2	F
C18POS	Glutamine	C5 H10 N2 O3	146.0691	0.86	5.680	0.475	0.768	**0.023**	**0.091**	0.348	0.537	1	F
C18NEG	Pyroglutamic acid	C5 H7 N O3	129.0425	0.93	3.138	0.149	0.482	**0.032**	0.119	0.678	0.446	1	F
HILICPOS	Betaine	C5 H11 N O2	117.0791	4.69	3.210	0.726	0.854	0.946	0.654	0.755	0.445	1	F
HILICPOS	Citrulline	C6 H13 N3 O3	175.0960	6.54	0.643	0.704	0.868	0.927	0.656	0.135	0.603	1	F
C18POS	Alanine	C3 H7 N O2	89.04767	0.86	1.814	0.792	0.788	0.502	0.539	0.455	0.434	1	G
HILICPOS	Glycerol 3-phosphate	C3 H9 O6 P	172.0139	6.62	1.056	0.086	0.447	0.519	0.538	**0.033**	0.447	1	G
C18POS	Creatine monohydrate	C4 H9 N3 O2	131.0694	0.89	1.798	0.942	0.821	0.564	0.532	0.170	0.524	2	G
C18POS	Anserine	C8 H16 N2 O3	188.1161	0.91	1.866	0.765	0.814	0.708	0.604	0.074	0.594	1	G
HILICPOS	N-Acetylcadaverine	C7 H16 N2 O	144.1265	4.11	2.433	0.689	0.891	0.206	0.388	0.346	0.554	1	G
HILICPOS	Ergothioneine	C9 H15 N3 O2 S	229.0889	5.67	2.277	0.089	0.386	0.531	0.533	0.353	0.488	2	G
C18POS	Sphingosine 1-phosphate	C18 H38 N O5 P	379.2485	5.84	3.127	0.750	0.843	0.518	0.546	0.393	0.438	1	H
HILICPOS	Aspartic acid	C4 H7 N O4	133.0375	6.77	5.898	0.890	0.832	0.217	0.386	0.950	0.419	2	H
HILICPOS	Glutamic acid	C5 H9 N O4	147.0534	6.39	1.528	0.322	0.674	0.247	0.404	0.281	0.563	1	H
C18POS	Phosphoserine	C3 H8 N O6 P	185.0089	0.85	0.737	0.577	0.829	0.314	0.434	0.284	0.543	1	H
HILICPOS	Palmitoylcarnitine	C23 H45 N O4	399.3352	0.72	4.467	0.756	0.827	**0.023**	**0.096**	0.391	0.448	1	H

Continued

Table S2 – Continued

Column & Mode	Metabolite	Molecular Formula	Molecular Weight	RT (min)	RSD_{QC}	*P* value G	FDR G	*P* value C	FDR C	*P* value GxC	FDR GxC	MSI ID	Cluster
C18POS	Decanoylcarnitine	C17 H33 N O4	315.2415	5.03	2.328	0.073	0.474	**2.28E-04**	**0.003**	0.594	0.441	1	H
C18POS	Hexanoylcarnitine	C13 H25 N O4	259.1786	3.7	4.698	0.090	0.369	**7.66E-05**	**0.002**	0.430	0.466	2	H
C18POS	Acetylcarnitine	C9 H17 N O4	203.1157	0.92	3.373	1.000	0.835	0.072	0.214	0.350	0.520	1	H
C18POS	Propionylcarnitine	C10 H19 N O4	217.1315	1.41	3.401	0.716	0.869	**0.035**	0.120	0.140	0.563	1	H
C18POS	Tryptophan	C11 H12 N2 O2	204.0901	3.19	2.013	0.809	0.795	**4.03E-04**	**0.004**	0.359	0.480	1	H
HILICPOS	S-Adenosylmethionine	C15 H22 N6 O5 S	398.1381	7.58	1.170	0.417	0.703	**0.010**	**0.043**	0.511	0.436	2	H
C18NEG	D-Ribulose 5-phosphate	C5 H11 O8 P	230.0188	0.79	4.838	0.696	0.871	**3.83E-05**	**0.001**	0.097	0.553	1	H
HILICPOS	Citicoline	C14 H26 N4 O11 P2	488.1081	7.86	3.003	0.869	0.843	**0.002**	**0.009**	0.377	0.445	2	H
C18POS	Adenosine	C10 H13 N5 O4	267.0967	1.02	5.060	0.172	0.512	**1.13E-04**	**0.002**	0.595	0.434	1	H
C18NEG	Xanthine	C5 H4 N4 O2	152.0332	1.01	5.299	0.333	0.680	**0.001**	**0.005**	0.444	0.434	1	H
C18POS	Hypoxanthine	C5 H4 N4 O	136.0384	1.02	6.369	0.366	0.676	**0.001**	**0.004**	0.687	0.437	1	H
C18NEG	Inosine	C10 H12 N4 O5	268.0805	0.92	2.479	0.221	0.535	**0.001**	**0.004**	0.835	0.440	1	H
HILICPOS	Inosine-5'-monophosphate (IMP)	C10 H13 N4 O8 P	348.0477	6.93	0.916	0.516	0.800	**0.008**	**0.036**	0.817	0.455	2	H

Table S2 – A total of 92 mouse gastrocnemius muscle metabolites were identified/annotated, including 16 metabolites that were significantly different between conditions (i.e., exercise versus rest) and one metabolite (N6,N6,N6-Trimethyl-L-lysine) significantly different between genotypes (i.e. DKI versus WT) based on univariate analysis. Statistically significant values ($P < 0.05$ and/or FDR < 0.1) appear in bold text. C: condition; G: genotype; G x C: interaction between condition and genotype; FDR: false discovery rate; MSI: Metabolomics Standards Initiative; RSD_{QC}: relative standard deviation in quality control samples; RT: retention time. Metabolites in this table are sorted by cluster.

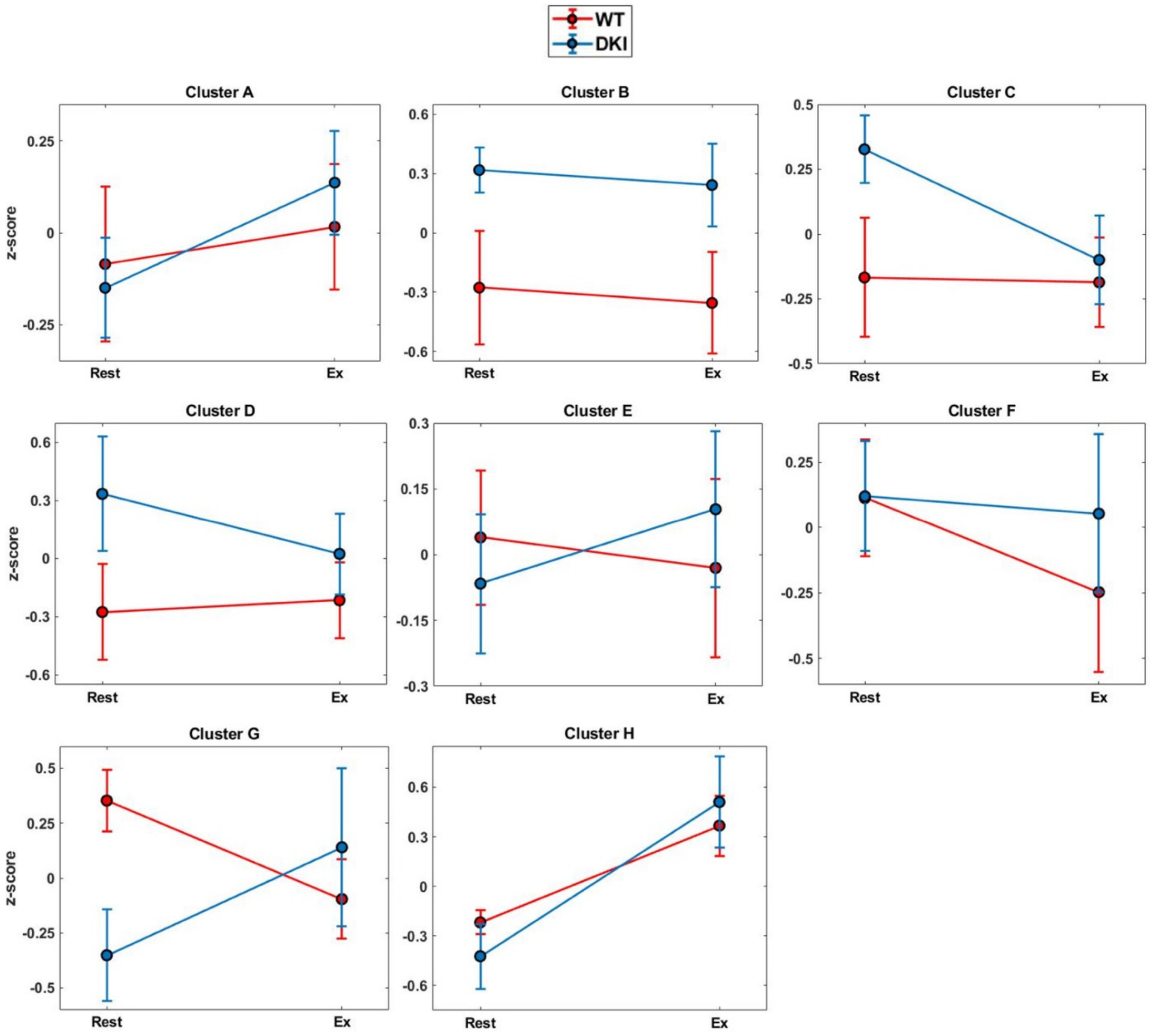

Figure S3 – Z-scores plot of the mean responses for each mouse gastrocnemius muscle metabolite cluster. After conversion of individual metabolite $\log_{10}$ responses to a z-score, the average response of each cluster was calculated and presented here as a group mean with error bars indicating the standard error (Red = WT; Blue = DKI). Following two-way ANOVA, Cluster B showed a significantly increase in abundance in DKI relative to WT mice ($P < 0.01$), independent of condition. Cluster B metabolites showed no significant association with exercise. Conversely, Cluster G demonstrated a significant interaction between genotype and condition ($P < 0.05$), with opposite metabolite trajectories observed between DKI and WT mice following exercise. Cluster G metabolites were decreased after exercise in WT mice while they were increased in DKI mice. Cluster H showed significantly increased metabolite abundance associated with exercise ($P = 3.58 \times 10^{-4}$) in both genotypes.

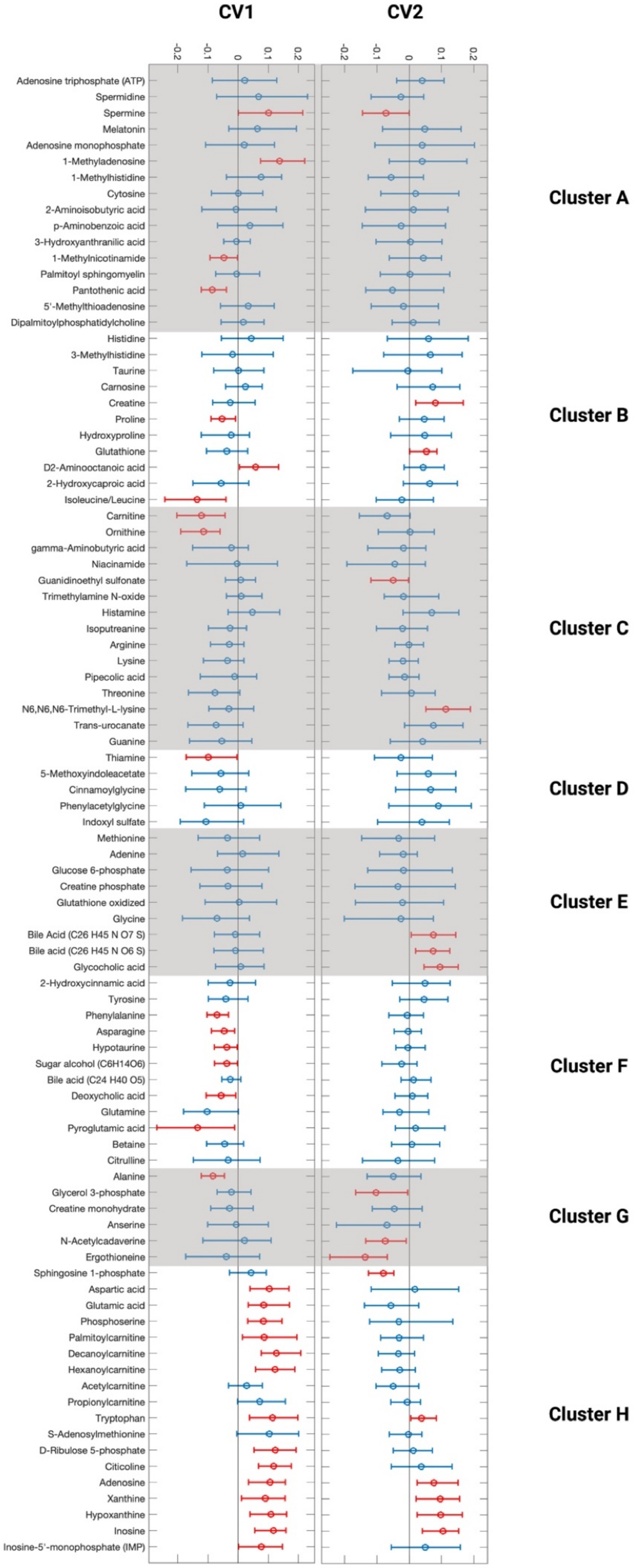

Figure S4 – Related to **Figure 5.** Loading plot showing mouse gastrocnemius muscle metabolites that significantly contributed to CV1 and CV2. Data are represented as mean loading values within 95% CI. Metabolites that significantly contributed to CV1 and/or CV2 are represented by red-colored dots and CIs, while blue dots and CIs represent metabolites that did not significantly contribute to their respective CV. Metabolites are sorted according to HCA clusters A to H (top-down). CI: confidence interval, CV: canonical variate, HCA: hierarchical cluster analysis.
